# DNA Adductomics for the Biological Effect Assessment of Contaminant Exposure in Marine Sediments

**DOI:** 10.1021/acs.est.3c00499

**Published:** 2023-06-21

**Authors:** Giulia Martella, Elena Gorokhova, Pedro F. M. Sousa, Natalia Y. Tretyakova, Brita Sundelin, Hitesh V. Motwani

**Affiliations:** † Department of Environmental Science, Stockholm University, SE-106 91 Stockholm, Sweden; ‡ Department of Materials and Environmental Chemistry, Stockholm University, SE-106 91 Stockholm, Sweden; § Department of Medicinal Chemistry and Masonic Cancer Center, University of Minnesota, Minneapolis, Minnesota 55455, United States

**Keywords:** DNA adducts, high-resolution mass spectrometry, biological effect monitoring, environmental contaminants, biomarkers, amphipods as sentinel species

## Abstract

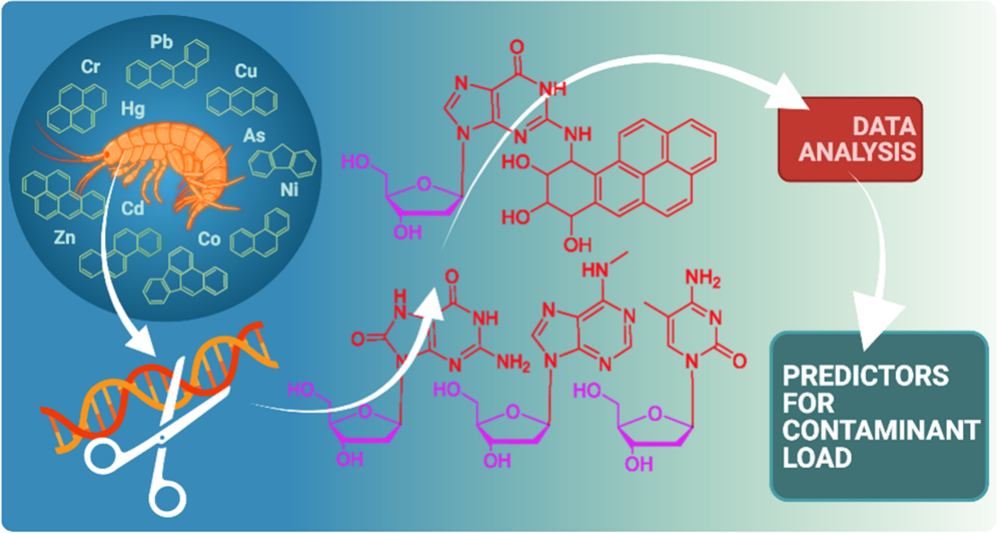

Exposure to chemical pollution can induce genetic and epigenetic alterations, developmental changes, and reproductive disorders, leading to population declines in polluted environments. These effects are triggered by chemical modifications of DNA nucleobases (DNA adducts) and epigenetic dysregulation. However, linking DNA adducts to the pollution load *in situ* remains challenging, and the lack of evidence-based DNA adductome response to pollution hampers the development and application of DNA adducts as biomarkers for environmental health assessment. Here, we provide the first evidence for pollution effects on the DNA modifications in wild populations of Baltic sentinel species, the amphipod *Monoporeia affinis*. A workflow based on high-resolution mass spectrometry to screen and characterize genomic DNA modifications was developed, and its applicability was demonstrated by profiling DNA modifications in the amphipods collected in areas with varying pollution loads. Then, the correlations between adducts and the contaminants level (polycyclic aromatic hydrocarbons (PAHs), trace metals, and pollution indices) in the sediments at the collection sites were evaluated. A total of 119 putative adducts were detected, and some (5-me-dC, N^6^-me-dA, 8-oxo-dG, and dI) were structurally characterized. The DNA adductome profiles, including epigenetic modifications, differed between the animals collected in areas with high and low contaminant levels. Furthermore, the correlations between the adducts and PAHs were similar across the congeners, indicating possible additive effects. Also, high-mass adducts had significantly more positive correlations with PAHs than low-mass adducts. By contrast, correlations between the DNA adducts and trace metals were stronger and more variable than for PAHs, indicating metal-specific effects. These associations between DNA adducts and environmental contaminants provide a new venue for characterizing genome-wide exposure effects in wild populations and apply DNA modifications in the effect-based assessment of chemical pollution.

## Abstract 2

DNA adductome analysis identifies exposure to environmental contaminants in a sentinel species in the Baltic Sea.

## 1 Introduction

Chemical pollution is one of the global anthropogenic pressures impacting wildlife. Like many other estuaries, the Baltic Sea is severely polluted due to human activities. There is a consensus that effect-based methods are needed to assess the toxicity of chemical mixtures for wildlife protection, especially considering the recent shift from exposures to high concentrations of a relatively small number of chemicals to exposures to low concentrations of many.^1^ In ecotoxicology, biomarkers and bioindicators are commonly used to detect the adverse effects of hazardous substances across different levels of biological organization—molecular, cellular, organismal, and population levels—via diagnostics of early signs of disease and pathologies in field-sampled organisms (reviewed by Lionetto et al.^2^).

Genotoxicity biomarkers are commonly used to elucidate the effects of chemical and physical agents on genetic material or deoxyribonucleic acid (DNA), leading to alterations in the structural and functional integrity of the genome. These alterations can, therefore, be informative as early warning signals in molecular epidemiology and biomonitoring studies.^3^ In addition, DNA damage is ecologically relevant because it is implicated in many pathological processes with effects beyond a lifetime of a single individual, including the quality and population persistence of the offspring. Although many genotoxic compounds are regulated, new industrial compounds and pharmaceuticals continue to emerge as contaminants.^4^ Therefore, genotoxicity assessment is integral to evaluating risks and impacts on human health and the environment.^5^ Traditionally, methods for evaluating genotoxicity in different organisms included micronucleus test as an index of chromosomal damage, comet assay for detecting DNA strand breaks, and DNA adduct detection by ^32^P-post-labeling of bulky aromatic adducts deriving from complex mixtures of environmental pollutants.^6−9^ The latter method targets DNA adduct detection to screen for genotoxic exposure and the resulting covalent binding of an electrophilic chemical/metabolite to nucleophilic sites on the DNA by a nucleophilic substitution reaction.^8^ However, ^32^P-post-labeling is a nonselective method that provides no structural information and thus cannot be used to understand specific structural modifications involved in toxicity outcomes.

DNA adducts are modified 2′-deoxyribonucleosides in the genome where a chemical moiety covalently binds the carbon or nitrogen atoms of the purine and pyrimidine. These lesions can be formed via various pathways.^10−13^ Genotoxic chemicals, such as polycyclic aromatic hydrocarbons (PAHs) and aromatic amines, can be metabolically activated to electrophilic reactive metabolites, which react with DNA to form nucleobase adducts.^14^ Additionally, exposure to chemicals causing inflammation and oxidative stress can induce the formation of reactive oxygen species (ROS), which oxidize DNA to form adducts such as 8-oxo-7,-8-dihydro-2′-deoxyguanosine (8-oxo-dG).^15−17^ If not repaired, DNA adducts induced by environmental chemicals and ROS can lead to DNA polymerase errors, mutations, and genotoxic effects.^18,19^ Moreover, exposure to exogenous and endogenous chemicals can lead to epigenetic dysregulation, whereby DNA methylation and hydroxymethylation patterns can be disrupted, leading to changes in gene expression.^20,21^

Today, liquid chromatography–mass spectrometry (LC-MS) techniques are a mainstay for DNA adduct analysis.^19,22−24^ Many DNA modifications, such as the bulky PAH adducts and methylated and oxidized lesions, can be quantified in a single DNA sample.^25−27^ High-resolution accurate mass spectrometry (HRAM) data on DNA adducts available from, for instance, Orbitrap high-resolution mass spectrometry (HRMS) facilitates the determination of their chemical structure, which can help exposure diagnostics. However, a nontargeted analysis is needed when no suitable DNA adduct library for the studied system is available. Recently, we developed a novel software (*nLossFinder*)^27^ for the nontargeted detection of DNA adducts using data-independent acquisition (DIA) mass spectral analysis of enzymatically hydrolyzed DNA. This method detects the characteristic neutral loss 116.0473 Da of 2′-deoxyribose from molecular ions of the 2′-deoxyribonucleosides adducts, allowing for the global identification of all structurally modified nucleobases.

DNA adductomics using the neutral loss approach analyses the totality of DNA adducts. However, this approach does not allow the detection of adducts formed on the phosphodiester backbone of DNA,^29^ depurinated adducts,^30^ and cross-linked products, such as DNA–DNA or DNA–protein,^31^ in a genome. This new *-omics* technology provides a comprehensive DNA adductome characterization by screening for multiple DNA modifications resulting from biological responses to endogenous and exogenous exposures. The method can be used in (eco)toxicology to assess damage to the (epi)genome and identify stressors associated with the adduct formation.^25,28^ Moreover, the high-throughput protocols for DNA adductome and other biomarkers can be combined in experimental and field studies, such as environmental monitoring and screening. For example, the usefulness of combining DNA adductomics and embryo aberration analysis in the amphipod *Monoporeia affinis* was recently demonstrated for biological effect monitoring in the Baltic Sea.^25^

Here, we report the development and application of DNA adductomics as a part of the biological effect assessment focusing on environmental contaminants. The test organism, *M. affinis*, is a benthic amphipod common in the Baltic Sea and regional lakes. In the Swedish National Marine Monitoring Program (SNMMP), *M. affinis* is used to assess the biological effects of contaminants in soft-bottom sediments because the embryo development in this species is susceptible to chemical exposure, with the impact manifested as developmental aberrations observable in embryos dissected from gravid females.^32,33^ Such aberrations not only indicate the exposure to the contaminants present in the sediments but may also suggest genotoxic effects with possible impacts on recruitment and genetic erosion in the amphipod populations. Furthermore, as sudden and poorly understood declines in amphipod populations in coastal systems suffering from pollution, including the Baltic Sea, have been reported,^34,35^ genotoxicity as a possible driver must be addressed, which requires a reliable method.

The aims of the present study were to (1) develop a workflow for sample analysis and data processing for untargeted detection of DNA adducts; (2) compare the DNA adduct profiles of the amphipods collected from areas with relatively high and low pollution levels with a particular focus on PAHs and trace metals; and (3) identify the contaminants associated with specific adducts. The findings demonstrate how DNA adductome can be explored for integration into the monitoring and environmental assessment.

## 2 Material and Methods

### 2.1 Sampling Areas and Their Contamination Status

#### 2.1.1 Amphipod Collection and Sampling Sites

**Figure 1:**
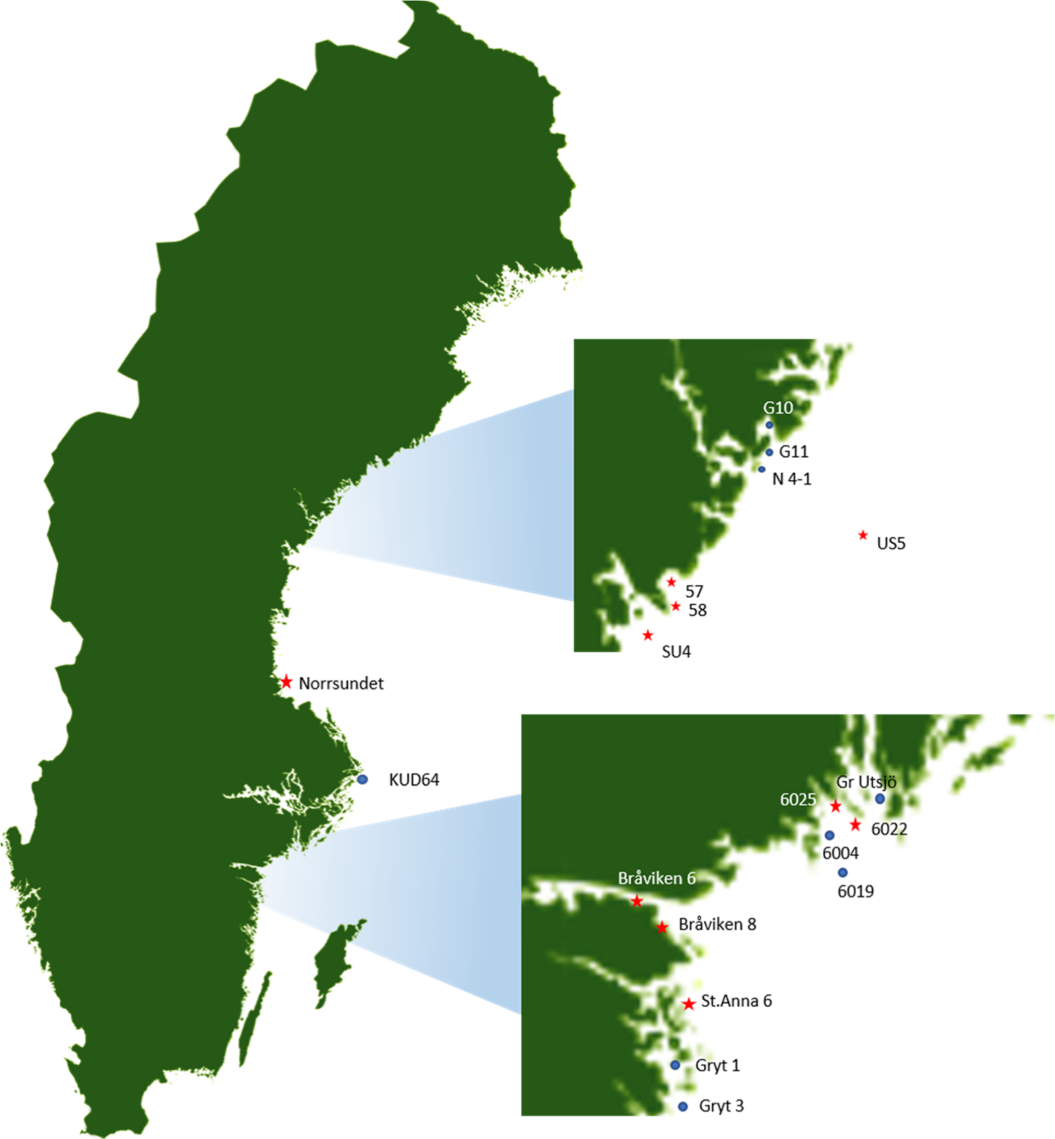
Sampling stations in the Bothnian Sea (top inset) and the Northern Baltic Proper (bottom inset) along the Swedish Baltic Sea coast. The stations with high contaminant levels (PAHs and metals) in the sediment are shown as stars, and those with contaminant concentrations below critical levels are shown as circles. The sites were classified as Contaminated and Reference, respectively; see Note S1 for details on the stations, contaminant concentrations, and the evaluation of the pollution status.

Gravid females of *M. affinis* were collected with a benthic sled at 19 stations with different pollution loads in the Bothnian Sea (BS) and the Northern Baltic Proper (NBP) as a part of the Effect Screening Study conducted by the Swedish Environmental Protection Agency in January 2018^36^ (Figure 1 and Table 1). The amphipods were transported in coolers (4–6 °C) with ambient sediment and water to the laboratory; the embryos were dissected, and the de-brooded females were frozen at −80 °C for DNA extraction and analysis.

**Table 1:** Summary for the Material and Contamination Status for All Sampling Stations in Each Basin (Bothnian Sea, BS, and Northern Baltic Proper, NBP), Number of Individuals Analyzed Per Station, Embryo Aberration Percentage in the Population (Based on 24–50 Individuals Analyzed, Data Are Available from ICES DOME Database; https://data.ices.dk)^a^

					contaminants		
basin	station	contaminated/reference	embryo aberrations, %	number of individuals	LmPAH (μg/kg dwt)	HmPAH (μg/kg dwt)	PLI (index)
BS	G 10	R	6.4	3	25	34	0.52
BS	G 11	R	5.4	2	26	56	0.97
BS	N 4-1	R	3.2	2	19	32	0.78
BS	US 5	C	6.9	1	80	314	2.01
BS	57	C	21.8	2	27 100	31 500	2.71
BS	58	C	11.7	2	34 100	43 100	2.16
BS	SU 4	C	8.9	2	573	557	1.99
BS	Norrsundet	C	10.7	10	626	397	0.78
BS	KUD 64	R	7.2	1	6	8	0.41
NBP	Gr Utsjö	R	11.2	2	58	99	0.76
NBP	6025	C	9.1	1	220	249	1.67
NBP	6022	C	8.1	2	135	218	1.28
NBP	6004	R	3.8	1	39	41	1.00
NBP	6019	R	5.3	1	5	8	0.35
NBP	Bråviken 6	C	8.6	4	97	157	1.41
NBP	Bråviken 8	C	10.2	4	201	310	1.64
NBP	St. Anna 6	C	8.5	4	247	488	1.47
NBP	Gryt 1	R	43.1	1	22	41	0.52
NBP	Gryt 3	R	9.4	2	115	151	0.41

a Based on the contaminant levels, the stations were classified as heavily polluted (Contaminated; C) and relatively unpolluted (Reference; R) as described in Note S1. The total concentrations of low- and high-molecular PAHs (LmPAHs and HmPAHs, respectively) and pollution level index (PLI) for the trace metals in the sediments were also calculated to provide integrated estimates.

#### 2.1.2 Contamination Level Assessment

The contaminant data were presented in our previous study relating benthic community structure to chemical pollution and eutrophication; see Raymond et al.^37^ for details. In brief, the sediment for chemical analyses was collected in 2011, 2012, and 2018 at the same sites as the test amphipods with a benthic sled set to sample the upper 2–3 cm of the sediment as described elsewhere.^33^ Thus, the measured concentrations represent the conditions in the uppermost sediment layer, where the amphipods usually reside. The macrofauna was removed by sieving with a 0.5 mm mesh, and the sediment was homogenized by stirring and frozen at −20 °C. The concentrations of PAHs and trace metals were analyzed as described in Note S1.

To classify the sampling sites into heavily polluted (Contaminated) and relatively unpolluted (Reference), we compiled information on the PAH and metal concentrations (Note S1 and Supporting File S1). Of the 19 stations that were sampled, 10 stations (57, 58, Norrsundet, Bråviken 6, Bråviken 8, St. Anna 6, 6022, 6025, US 5, SU 4) were assigned as contaminated, and nine stations (6019, 6004, Gryt 1, Gryt 3, Gr Utsjö, G 10, G 11, N 4-1, KUD 64) as reference sites.

### 2.2 DNA Adducts Analysis

#### 2.2.1 Chemicals and Other Materials

Deoxyribonucleic acid from calf thymus (ctDNA) sodium salt, 2′-deoxyguanosine (dG), 2′-deoxycytidine (dC), 2′-deoxyadenosine (dA), thymidine (T), 5-methyl-2′-deoxycytidine (5-me-dC), 8-oxo-7,8-dihydro-2′-deoxyguanosine (8-oxod-G), N^6^-methyl-2′-deoxyadenosine (N^6^-me-dA), nuclease P_1_ from *Penicillium citrinum* (NP1), phosphodiesterase I from *Crotalus adamanteus* (snake) venom (SVPDE), alkaline phosphatase from *Escherichia coli* (AKP), ammonium acetate, ammonium bicarbonate, tris(hydroxymethyl)aminomethane (Tris-buffer, pH 7.4), zinc chloride, and formic acid were obtained from Sigma-Aldrich (St. Louis, MO). Chelex-100 resin was purchased from Bio-Rad (Solna, Sweden). All solvents used were of HPLC grade. All experiments with DNA were carried out in DNA LoBind tubes, 1.5 mL (Eppendorf). N^2^-[-10-(7,8,9-trihydroxy-7,8,9,10-tetrahydrobenzo[*a*]pyrenyl)]-2′-deoxyguanosine (BPDE-dG) was available from an earlier synthesis.^38^

#### 2.2.2 Sample Preparation

DNA samples used in this work were isolated from 47 individual amphipods from the collection described in Section 2.1. The protocol for DNA extraction and the enzymatic cleavage described in our earlier work^25^ was employed. Individual samples were manually homogenized, and DNA was extracted using a suspension of Chelex-100, an ion exchange resin. Fifteen microgram DNA were used for the enzymatic digestion by nuclease P1 (NP1), snake venom phosphodiesterase I (SVPDE I), and alkaline phosphatase (AKP), which yielded the 2′-deoxyribonucleoside adducts and unmodified 2′-deoxyribonucleosides. The samples were stored at −20 °C until analysis by LC-HRMS/MS as described below. In addition, six individuals’ amphipods were processed in triplicates to evaluate the repeatability; three 15 μg aliquots of DNA from each animal were subjected to enzymatic digestion, LC-HRMS, and data processing.

#### 2.2.3 Liquid Chromatography

**Figure 2:**
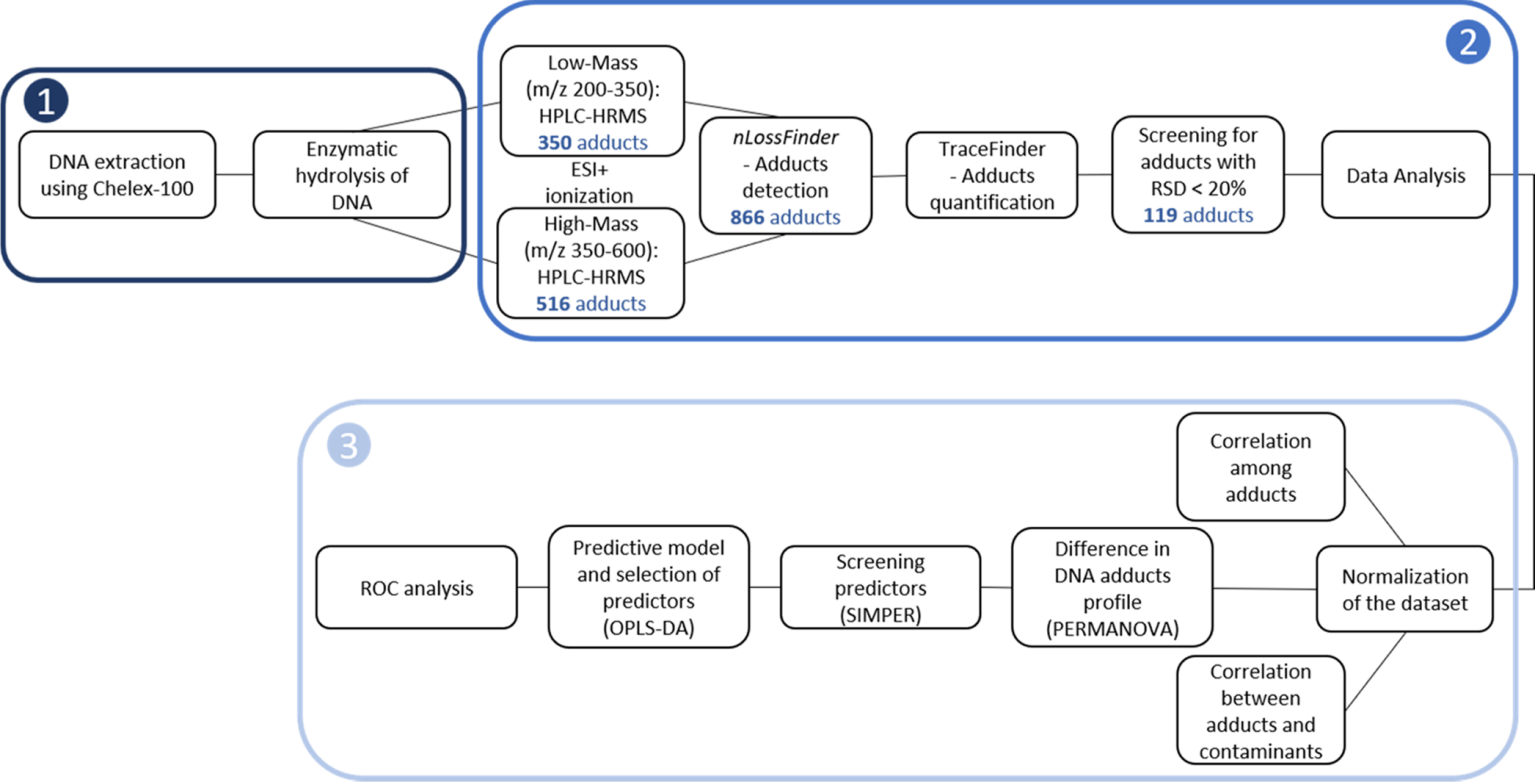
Workflow of sample preparation (1), HRMS analysis and data processing (2), and statistical evaluation (3) performing permutational multivariate analysis of variance (PERMANOVA), similarity percentage analysis (SIMPER), orthogonal partial least squares discriminant analysis (OPLS-DA), and receiver operating characteristic (ROC) analysis.

Each sample was injected twice for high-pressure liquid chromatography (HPLC) coupled to HRMS analysis, the first time using the *m/z* scan range from 195 to 355 and the second time using the 350–600 *m/z* range (Figure 2). The LC-MS system consisted of a Dionex UltiMate 3000 LC device interfaced to an Orbitrap Q Exactive HRMS (Thermo Fisher Scientific, MA). The mobile phase of the LC system consisted of a mixture of water–methanol; system A with 5% methanol/water and system B with 95% methanol/water, each containing 0.1% formic acid. The HPLC column was a Superlco Ascentis Express F5 2.7 μm HPLC column (15 cm × 2.1 mm) from Sigma-Aldrich. The HPLC injection volume was 10 μL, with 120 μL/min flow rate and a column temperature 25 °C. One LC gradient each was optimized and applied for the two scan ranges. The LC gradient used for low-mass adducts (scan range from 195 to 355 *m/z*) consisted of an initial 2 min equilibration at 5% of B, followed by a linear increase to 30% in 10 min and then to 100% in 4 min. After holding at 100% B for 3 min, the solvent composition returned to the initial condition of 5% B in 2 min, and the system was reequilibrated for 4 min before the next injection. The LC gradient applied for high-mass adducts (scan range from 350 to 600 *m/z*) consisted of 30 min gradient elution, starting with 5% B held for 2 min, followed by a linear increase to 30% B at 9 min and further to 100% B at 22 min. The solvent composition was held at 100% B for 6 min and returned to initial conditions, followed by equilibration for 2 min. For both gradients, an automated switch valve was connected between the LC column and the Orbitrap MS, which was set to allow the eluent from the column to enter the waste during the 1st minute after injection to divert polar impurities. After 1 min, the valve was switched back to allow the eluent to enter the ion source of the mass spectrometer.

#### 2.2.4 Orbitrap HRMS Analysis

HRMS analysis was conducted using an Orbitrap Q Exactive HF mass spectrometer equipped with a heated electrospray ionization (HESI) source. The optimized MS parameters were as follows: spray voltage, 3.5 kV; spray current, 22 μA; capillary temperature, 275 °C; sheath gas, 20 arbitrary units (au); auxiliary gas, 10 au; S-Lens RF level, 60%; and probe heater temperature, 240 °C. The MS was primarily operated in the positive ionization mode using a normalized collision energy of 30 eV. DNA adducts were screened as modified 2′-deoxyribonucleosides (dNs) using full MS/data-independent acquisition (DIA) mode. The full MS scan was conducted at a resolution of 120 000, automatic gain control (AGC) target 3e^6^, maximum ion injection time (IT) 200 ms, and scan range from 110 to 650 *m/z*.

For the low mass range run, DIA was set to a mass resolution of 60000, AGC target 5e^5^, maximum ion IT 120 ms, loop count 16, and scan range from 195 to 355 *m/z*, which was divided into 16 discrete *m/z* intervals, with an isolation window of 10 *m/z* (200 ± 5, 210 ± 5, and up to 350 ± 5 *m/z*). For the high-mass range run, the DIA was set to a mass resolution of 60000, AGC target 5e^5^, maximum ion IT 120 ms, loop count 26, and scan range from 347 to 607 *m/z*, which was divided into 26 discrete *m/z* intervals with an isolation window of 10 *m/z* (347 ± 5, 352 ± 5, and up to 607 ± 5 *m/z*).

#### 2.2.5 Processing of HRMS Data

Screening for putative 2′-deoxyribonucleoside adducts from the MS raw data was performed using the in-house-developed software *nLossFinder*.^28^ The parameters used for the low- and high-mass detection included tolerance of 5 ppm for the deoxyribose (dR) neutral loss mass (116.0473 Da), gauss sigma 0.075, noise filter width 8.0, zero area filter ZAF 1/ZAF 2 0.085/0.165, and signal-to-noise ratio 5. Eight raw data samples of digested DNA from 4 individuals, two from the contaminated and two from the reference areas, each for low and high-mass 2′-deoxyribonucleoside adducts, were screened using the *nLossFinder*. The output lists from these eight processing sets were combined into a single file to create a master list of putative DNA adducts. The resulting list of putative adducts was manually evaluated for peak quality. Subsequently, *TraceFinder* software (V4.1) from Thermo was used for confirmation of the peaks and to obtain individual peak areas (Figure 2). Possible ESI adducts and isotopes were not removed from the list. The program was set up to screen 350 putative adducts in the acquisition list within the low mass range and 516 putative adducts within the high mass range. The 2′-deoxyribonucleoside adducts were considered to be detected if precursor ions were found in *TraceFinder* with mass accuracy within 5 ppm of what was obtained by *nLossFinder* and at a comparable retention time. The elemental composition of the influential adducts (cf. Section 3.3) was predicted using Thermo Xcalibur Qual Browser. Minimum numbers of C, O, and N atoms were set as 9, 3, and 2, respectively, with charge 1 and mass tolerance 5 ppm.

In each sample, the peak areas of each putative adduct obtained from *TraceFinder* were normalized to the peak area of dG (peak area-adduct *100/peak area-dG). Similarly, normalized peak areas were obtained for the six amphipod DNA digested and analyzed in triplicates. The relative standard deviation (RSD) was estimated using normalized peak areas from the triplicate subsamples; the 2′-deoxyribonucleoside adducts with RSD below 20% were used for statistical evaluations, assuming equal MS response (see Section 2.3).

### 2.3 Data Analysis and Statistics

Two-tailed hypothesis tests were applied for all statistical comparisons, with *p* < 0.05 considered significant. The numerical variables were assessed for normality using the Shapiro–Wilk test, and the numerical and qualitative variables were summarized using descriptive statistics. The analysis was run in R^39^ and the online platform *MetaboAnalyst*.^40^

The data on the individual peak areas for the 2′-deoxyribonucleoside adducts were log-transformed^41^ to stabilize the distribution and normalized by log(dG)^25,42^ to adjust the sample losses during preparation and instrument drift between the runs. Further, Pareto scaling with log transformation was applied on the log(dG)-normalized peaks to reduce the influence of outliers using the R package *IMIFA*. Finally, the contaminant concentrations were log-transformed and scaled to their *z* scores by subtracting the mean value and dividing by the standard deviation. The adducts and chemical concentrations that were invariant across the dataset were excluded from further analysis.

First, we used Pearson correlation analysis to explore cross-correlations among the adducts (transformed and normalized data) and univariate correlations between the adducts and single contaminant concentrations (log-transformed and scaled values). The correlations were presented as heatmaps for Pearson *r* and the associated *p* values using the R packages *gplots*, *corrplot*, and *High-massisc*.

Second, the differences in the DNA adduct profiles between the heavily contaminated and the reference sites and between the basins were evaluated with Contamination status (contaminated vs reference sites) and Basin (Bothnian Sea vs Baltic Proper) as categorical factors. Initially, the effects of Contamination status, Basin, and their interaction were evaluated by a nonparametric, permutational multivariate analysis of variance (PERMANOVA, Table S6) with 999 permutations, fixed effects summed to zero, Type III sum of squares, and 5% significance level as implemented in the R package *vegan*. The function *betadisper*, from the same package, was used for the Permutation test for homogeneity of multivariate dispersions (PERMDISP). This approach addresses the whole DNA adduct profile responses while accounting for possible interdependencies between the adducts and potential nonlinear relationships; it is robust to be used with correlated measurements and suitable for data that do not have an identifiable distribution.^43^

Third, when a significant Contamination status effect was detected, an orthogonal partial least squares analysis model (OPLS-DA), as implemented in *MetaboAnalyst*,^40^ was used to identify the most influential adducts for the overall discrimination ability between the contaminated and reference sites. As our dataset contained a relatively low number of observations (47) and a high number of adducts (119), the overfitting with PLS-DA was likely. Therefore, SIMPER analysis was applied to select adducts responsible for 80% of the dissimilarities using the R package *vegan* and thus decrease the number of predictors. The selected adducts were used as predictors to classify the sites as contaminated/reference in the OPLS-DA model, with 10-fold cross-validation and *Q*^2^ as a performance metric. The influential adducts were selected based on their variable importance in projection (VIP) values accounting for Component 1. Predictor confirmation was evaluated using the area under the receiver operating characteristic (ROC) curve (AUC), *t*-test, and fold-change analysis. See Figure 2 for the workflow used for the sample preparation and the data analysis.

## 3 Results

### 3.1 nLossFinder Screening and DNA Adduct Profiling

**Figure 3:**
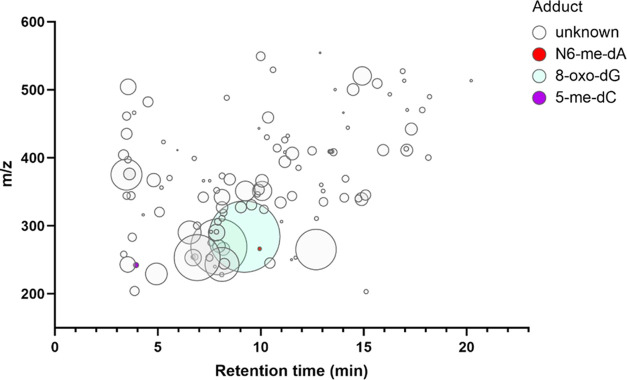
Example of a DNA adductome map representing modified nucleobases detected in genomic DNA using the untargeted *nLossFinder* approach followed by *TraceFinder* processing. The *X*-axis shows the HPLC retention times of detected 2′-deoxyribonucleoside adducts, and the *Y*-axis shows the *m*/*z* values of their precursor ions (200–600 range). Circle size represents the relative abundance of the adducts (i.e., the peak area of the adduct normalized to dG). The DNA originated from an individual inhabiting highly contaminated sediments (station Bråviken 6). Structurally identified adducts confirmed by standards are marked in color. 2′-Deoxyribonucleoside adducts with a low mass range have an elution time between 1 and 15 min, with a major cluster eluting in 6–10 min intervals, where the mobile phase mainly consists of water, implying that low-mass adducts are more hydrophilic. Adducts detected at a retention time of 15–25 min are more hydrophobic as they elute toward the end of the gradient program, where the mobile phase mainly consists of methanol.

Untargeted screening for 2′-deoxyribonucleoside adducts by *nLossFinder*, using four representative amphipods, resulted in 350 putative 2′-deoxyribonucleoside adducts in the low mass range and 516 putative adducts in the high mass range (Figure S5). The data were filtered using *TraceFinder* to eliminate plausible false positives. Moreover, adducts with high between-replicate variability (RSD% > 20%) for the triplicate samples used for the method evaluation were excluded. This procedure yielded 119 adducts (Figure 3) in the mass range of 200–600 *m/z* (Figure 3), representing a fingerprint of all DNA modifications in a specimen.

The DNA adductome map (Figure 3) shows that most 2′-deoxyribonucleoside adducts are eluting in the first 10 min of the HPLC run, with only a few appearing between 15 and 25 min. Examples of the low-mass adducts include 5-me-dC (L58), *N*^6^-me-dA (L129), and 8-oxo-dG (L167), which were confirmed by their accurate mass and comparison of HPLC retention times with their respective reference standards. The high-mass PAH adduct BPDE-dG used as a reference compound for the LC method development for the high-mass adducts (Note S2) was not detected in these samples. Supporting File S2 summarizes the characteristics of all of the measured 2′-deoxyribonucleoside adducts in terms of their retention time and *m/z* values observed for molecular- and fragment-ions with high mass accuracy.

### 3.2 Correlations between the Adducts and Specific Contaminants

Pearson cross-correlations for the detected adducts revealed positive within-group correlations, i.e., high-mass adducts associated with other high-mass adducts and low-mass adducts associated with other low-mass adducts (Figure S7). Several adducts, e.g., H113/H111, H7/H6, L127/L123, L222/L189, L64/L60, and L69/L94, had correlation coefficients close to 1 (*r* = 0.98–0.99). However, none of these strongly correlated adducts had the same chromatographic retention, ruling out the possibility that these correlations are due to potential ESI adducts or isotopes.

**Figure 4:**
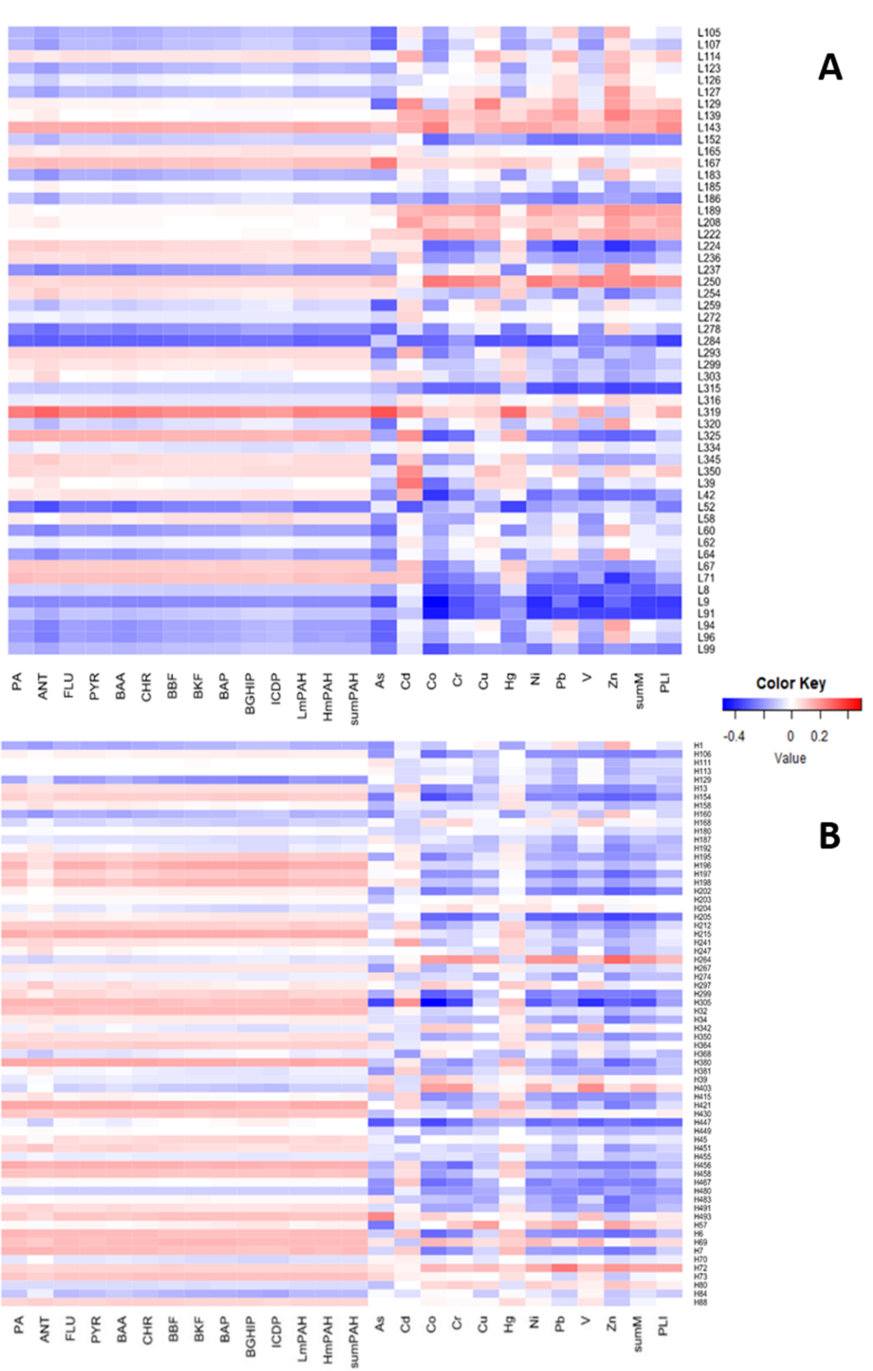
Heatmap of Pearson correlations between the relative abundance of the adducts and contaminant concentrations for the low-mass (A) and high-mass adducts (B). The color shade indicates the strength (weak/strong), and the color indicates the direction of the correlation (positive/negative).

Pearson correlations between the adducts and contaminant concentrations in the sediment indicated different patterns for PAHs and metals. The correlations with PAHs were similar across the congeners, resulting in a relatively homogeneous pattern across all 11 congeners, with only a few significant correlations between the PAHs and low- and high-mass adducts (Figures 4 and S8). However, the high-mass adducts were significantly more likely to correlate positively with the PAHs than the low-mass adducts (*t*-test; *p* < 0.004 in all cases; Figure S9 and Table S7). In contrast, correlations between the adducts and metals were often metal-specific, with many significant correlations for both low- and high-mass adducts (Figures 4 and S8). Moreover, several adducts showed both positive and negative correlations with metals (Figure 4). For example, L319 had negative correlations with Pb and Zn and positive correlations with other metals (As, Cd, Co, Cr, Cu, Hg, Ni, and V), and L39 had positive correlations with Cd, Cu, and Hg and negative correlations with other metals (As, Co, Cr, Ni, Pb, V, and Zn).

### 3.3 Identification of Influential Adducts

**Figure 5:**
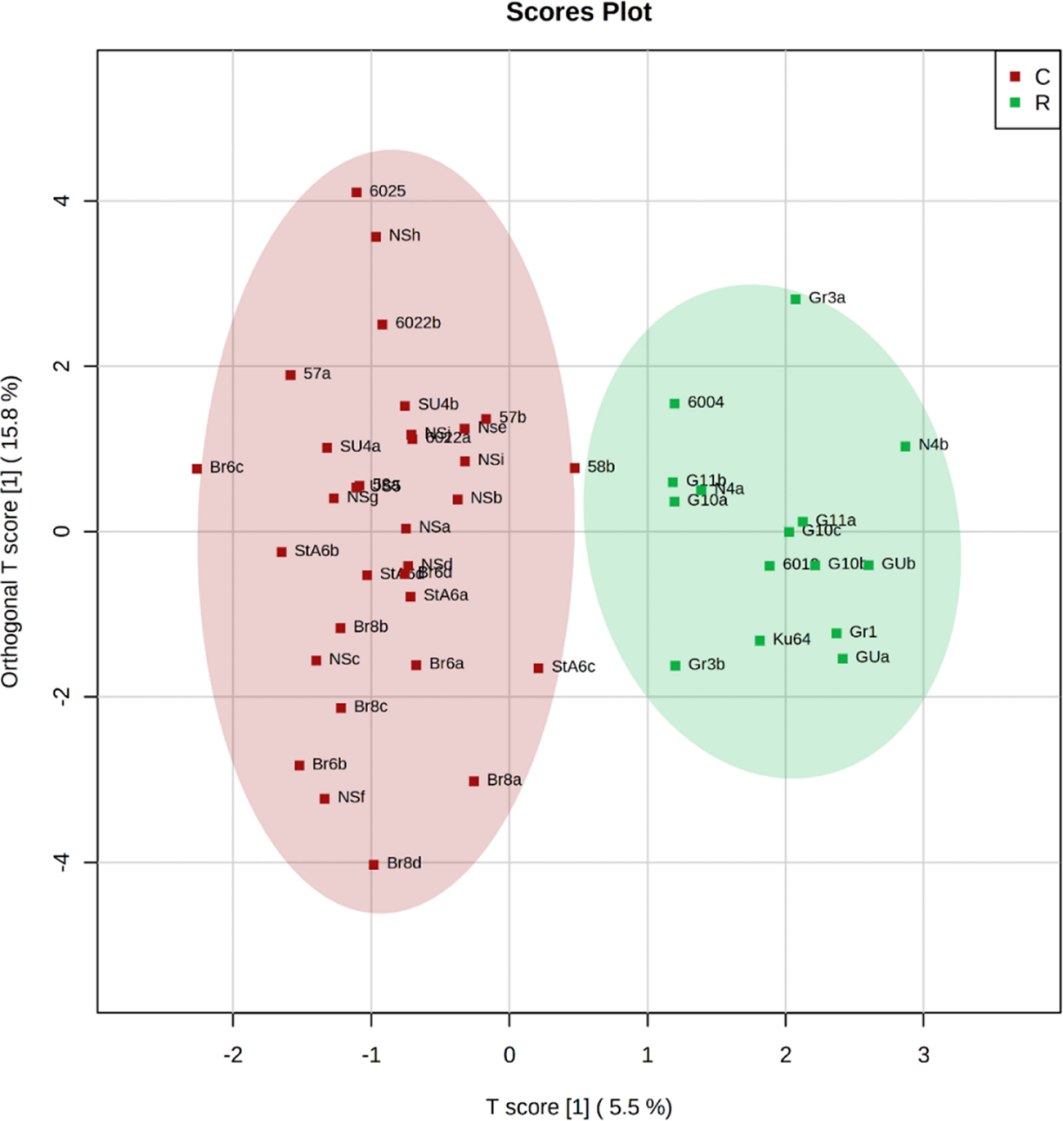
Score plot of OPLS-DA. The data points represent the samples collected in the stations classified as contaminated (red) or reference (green).

When comparing the DNA adductome profiles between the contaminated and reference sites, SIMPER analysis identified 37 adducts responsible for 80% of the dissimilarity between the datasets for the contaminated (10 stations, 28 individuals) and reference (9 stations, 19 individuals) areas (Supporting File S3). Using these adducts as possible predictors, we generated a significant OPLS-DA model with a clear separation between the sites (Figure 5, *Q*^2^ = 0.40 and *R*^2^*Y* = 0.85).

**Figure 6:**
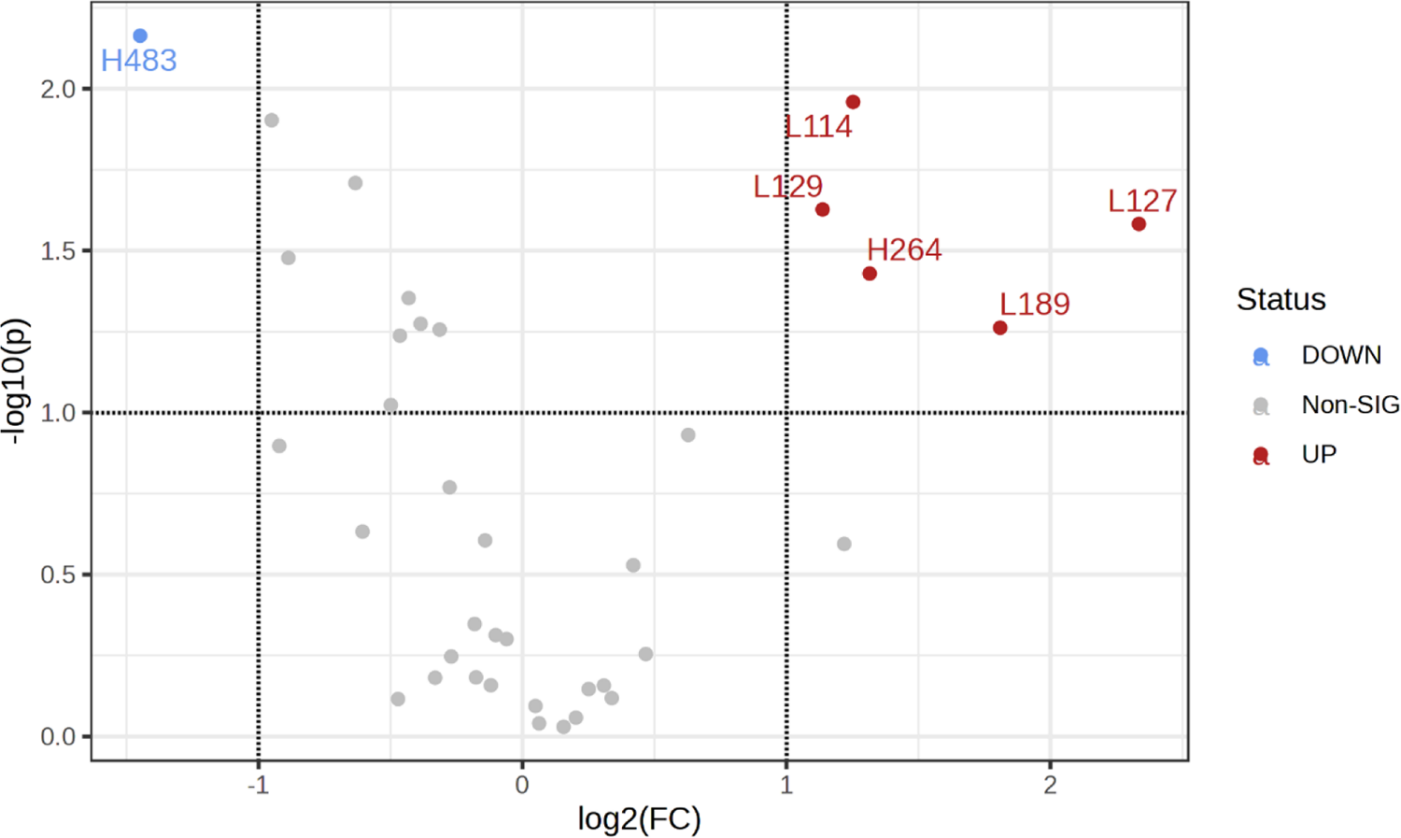
Volcano plot showing the adducts that were significantly upregulated (red) and downregulated (blue) in the contaminated sites compared to the reference sites. The data include the 37 putative adducts used for the statistical analysis following the SIMPER screening. The *X*-axis represents the log_2_ fold change (FC) with a threshold of 2; the direction of the comparison is Contaminated/Reference. The *Y*-axis is −log 10(*p*) with a *p*-value threshold of 0.1.

Both low- and high-mass adducts were important for the first component (Figure S10), with significantly downregulated H483 and upregulated L114, L127, L129 (N^6^-me-dA), L189 and H264 in the contaminated areas (*t*-test; Figure 6 and Table S8). Using the selected features H483, L189 and L114, the ROC analysis showed the best discrimination capacity with an AUC value of 0.82 (95% CI: 0.641–0.959; Figures S11 and S12). Moreover, the elemental composition of the adducts identified as influential by the OPLS-DA model was proposed (Table 2).

**Table 2:** Adducts Identified as Significant Predictors for Discriminating Between the Contaminated (C) and Reference (R) Sites According to the OPLS-DA Model^a^

putative adduct	precursor ion observed, *m/z*	product ion observed, *m/z*	retention time, min	proposed elemental composition of nucleoside adduct	mass accuracy, Δppm	RDB	C/R
H483	529.2973	413.2512	10.6	C22 H44 O12 N2	1.1	2.0	↓
H187	406.2178	290.1712	11.5	C17 H31 O8 N3	–1.4	4.0	↓
L114	258.0947	142.0477	3.30	C9 H13 O5 N4	–1.2	5.5	↑
L127	266.0822	150.0357	8.20	not determined			↑
H342	459.2450	343.1968	10.3	C20 H34 O8 N4	0.1	6.0	↓
L129(N^6^-me-dA)	266.1242	150.0776	9.90	C11 H14 O3 N5	–2.1	7.5	↑
H403	482.2248	366.1769	4.50	C21 H31 O8 N5	0.5	9.0	↓
H368	466.2640	350.2189	14.0	C20 H33 O5 N8	–1.4	8.5	↓
L189	291.0887	175.0419	7.80	not determined			↑
H264	430.1841	314.1348	10.2	C18 H27 O9 N3	4.8	6.5	↑
H204	411.2129	295.1653	17.1	C20 H31 O7 N2	0.7	6.0	↓

a Chemical characteristics assigned to the DNA adducts were the exact mass for the precursor ion (i.e., 2′-deoxyribonucleoside adduct) and the product (nucleobase adduct) ions, [M + H]^+^ and [(M – dR) + H]^+^, respectively; the retention time under the employed LC conditions; the proposed elemental composition; mass accuracy (Δppm) determined as a difference between the parent ion mass observed and that calculated using the predicted elemental composition; and the possible number of double bonds (RDB). Upregulated (↑) or downregulated (↓) change in the relative abundance of the adduct as characteristic of the contaminated (C) in relation to the reference (R) group is also shown (C/R).

## 4 Discussion

DNA adductomics approach was recently introduced as a new effect-based tool for predicting the ecotoxicological effects of chemicals in the aquatic environment.^25^ Here, we report an important development and environmental application of DNA adductomics using HRMS to detect genomic DNA adducts within a defined mass range. After developing the workflow and implementing the untargeted adduct detection, we compared the amphipod DNA adductome between the areas with relatively high and relatively low PAH and trace-metal levels in the sediments. We found significant variability in the DNA adductome of animals collected from marine sediments with different pollution loads, identified the adducts driving these differences, and revealed the contaminants associated with specific adducts. This is the first study to report associations between DNA adducts and environmental contaminants in the context of effect-based methods and demonstrate how DNA adductome can be explored for integration into the monitoring and effect assessment of hazardous substances.

### 4.1 Method Development and Adducts Detection

The overall workflow (Figure 2) with improved software-assisted detection allowed us to analyze the mass spectrometry data for potential novel 2′-deoxyribonucleoside adducts and generate a list of potential DNA adduct candidates. A comprehensive nontarget approach was applied using the software *nLossFinder*, developed for DNA adduct screening. The first screening using the *nLossFinder* provided a summary of the characteristics of all of the measured 2′-deoxyribonucleoside adducts, the retention time, *m/z* values observed of molecular ions and nucleobase fragments with high mass accuracy, which can facilitate obtaining more structural information of the individual adducts, for instance, by performing targeted MS2 or MS3 experiments. Subsequent semiquantification based on normalized peak areas provided the final selection of the adducts that could be linked to the stress factors, such as chemical contamination.

Our findings confirmed the detection of the DNA adducts investigated in our previous work on *M. affinis*.^25^ The two epigenetic marks [5-me-dC (L58) and N^6^-me-dA (L129)], the oxidatively generated adducts [8-oxo-dG (L167), 5-OH-dC (L63) and 8-OH-dA (L134)] and the deaminated 2′-deoxyribonucleosides [dI (L91) and dU (L35)] were detected by *nLossFinder* and consequently integrated by *TraceFinder*. Putative DNA adducts were selected to examine whether the variation of the instrument response for three replicates had RSD% < 20 and ensure the digestion and MS detection repeatability. We have identified a total of 119 adducts, which included 53 low-mass adducts and 66 high-mass adducts. Within the candidate adducts, the acceptable RSD were found for 5-me-dC (L58), N^6^-me-dA (L129), 8-oxo-dG (L167), and dI (L91) that were then included in the list of adducts used for the data analysis. Since all samples were treated identically, any potential effects of artifactual oxidation^44^ of deoxyribonucleosides should be uniform, with negligible effects on the between-sample variability. We are currently developing an in-house adductomics database focused on the environmental domain, which will include common and IUPAC names, chemical formulas, MS1 and MS2 spectra information, and relevant metadata (depending on availability). As much as possible, it will be integrated with existing databases [e.g., Guo et al.^45^ and La Barbera et al.^46^].

### 4.2 Associations between Adducts and Contaminants

According to the current regulations,^47^ PAHs and several metals (e.g., Cd, Zn, and Hg) exceeded their safe levels in the contaminated sites, with metals contributing most to the differences between the contaminated and reference sites (Figure S4). Both contaminant groups may affect DNA by (i) inducing ROS and oxidizing the nucleobases,^17^ (ii) inhibiting or promoting the activity of enzymes responsible for the regulation of DNA methylation and demethylation,^48,49^ and (iii) altering the rates of DNA deamination, e.g., deamination of exocyclic amines of DNA, which is a form of DNA damage linked with nitrosative stress and deaminase enzyme activity.^50^ PAHs can also affect DNA by covalent binding of their reactive metabolites to nucleophilic sites of nucleobases.^14,51^

Overall, the univariate correlations between the sediment PAH concentrations and DNA adducts were significantly higher for the high-mass than for the low-mass adducts (Figure S9 and Table S7). The high-mass adducts showed mostly weak univariate positive correlations with PAHs (Figure S8), possibly due to PAH-DNA adduct formation. Few significant correlations for the adducts L284, L319, L52, and H129 with PAHs were also observed (Figure 4). As a result of bioactivation, PAHs can form DNA adducts, with a mass in the range *m/z* 350–600.^22,52^ Exposure to PAHs could lead to an increased formation of ROS or other reactive intermediates that can induce, for example, lipid peroxidation and subsequent formation of DNA adducts.^53^ Once the structures of the PAH-correlated high-mass adducts are identified, it will be possible to know if bioactivation occurs in the amphipods or if the high-mass adducts are a product of endogenous processes leading to, e.g., lipid peroxidation adducts. The negative correlations observed are likely not originating from the covalent binding of specific PAHs with DNA forming PAH-DNA adduct. Moreover, the fact that the correlations with PAHs are uniform across the congeners for both low- and high-mass adducts might indicate that these congeners act additively. In mixtures, PAHs induce toxicity following the concentration-addition model;^54^ therefore, the chemical activity concept that reflects the mixture potential to cause baseline toxicity^55^ might be a way to evaluate the occurrence of PAH-DNA adducts in the contaminated environments.^56^ In addition, similar correlations for Hg and PAHs in all low-mass and most high-mass adducts (Figure 4) indicate possible joint effects of Hg and PAHs on DNA.^57,58^

Both positive and negative correlations between the adducts (L284, L315, L319, L8, L9, L91, H154, H205, H299, H305, and H447) and metals (As, Co, Zn, Pb, Cr, Hg, and PLI), suggest effects of metal exposure on DNA modifications. As no significant negative correlations were observed among these metals (Figure S3), the multicollinearity as a reason for both positive and negative correlations can be ruled out. Genomic levels of some adducts [L284, L9, dI (L91), H305, and H447] were not significantly different between the animals from contaminated and reference sites (Figure S10), but they had significant correlations with some metals, making these adducts potentially valuable for monitoring. Moreover, Co, As, and Zn correlated significantly with several adducts, underlying the importance of metal exposure to the dissimilarities in the adduct profile between the Contaminated and Reference sites (Figure S4). In particular, dI (L91) showed negative correlations with several metals (Co, Cr, Ni, Pb, V, and Zn). dI is a product of the deamination of dA via several mechanisms, including enzymatic reaction with adenosine deaminase (ADA).^59^ Heavy metals can inhibit the ADA enzyme,^60,61^ possibly explaining the observed negative correlations between dI and these metals. Similar mechanisms could be responsible for the other negative correlations between the metals and the adducts.

### 4.3 Epigenetic Modifications

The methylated 2′-deoxyribonucleoside N^6^-me-dA (L129) was a significant predictor for the contaminant exposure suggesting the importance of the epigenetic component in the response. This adduct can be formed enzymatically by specialized DNA methylases or via chemical reactions of methylating agents with DNA or the nucleotide pools.^62,63^ However, N^6^-me-dA has been widely detected in bacterial DNA, including *E. coli*,^64^*Mycoplasma*,^65^ and the microbiome of nematodes.^66,67^ As we used whole bodies of the amphipods for the DNA extraction, it cannot be ruled out that at least some of the detected N^6^-me-dA originated from the animal microbiota or bacterial food associated with sediment and not the host DNA. It is thus essential to evaluate the relative contribution of the bacterial N^6^-me-dA to the total pool of the adduct detected at the holobiont level if we are to use this marker for environmental diagnostics in selected indicator species. Future studies should address bacteria-specific N^6^-me-dA content by isolating bacterial DNA associated with the host and using it for the analysis of DNA modifications.

DNA methylation and demethylation processes can be affected by environmental contamination.^48^ Both metals and PAHs (such as benzo[*a*]pyrene) are capable of inhibiting or promoting the DNA methylation pathways.^68,69^ Exposure to Cd, Hg, or Pb can alter the DNA methylation pattern,^48^ by affecting the activity of the DNA methyltransferase (DNMT) enzyme. Global DNA methylation can be assessed by DNA hydrolysis followed by LC-MS/MS analysis, and several studies on the epigenetic marks, such as 5-me-dC, demonstrated the accuracy of the methylation assessment using this approach.^21,70^

### 4.4 Value for Monitoring and Assessment

In our study, delineating contaminated and reference sites was challenging because no sediments that could be qualified as unpolluted were present in the dataset, which is a rather typical situation in estuarine systems with high mixing and horizontal transport of pollution resulting in many contaminants occurring far away from the emission sources.^71,72^ Therefore, it is unsurprising that the PLS model based on the DNA adduct profiles, albeit significant, has a relatively low *Q*^2^ (0.4; Figure S10), indicating that model predictability can be improved by including more animal samples from relatively unpolluted sites in the dataset. Alternatively, animals raised in the laboratory under controlled conditions can be used to represent DNA adduct profiles for unpolluted environments.

The field data obtained in this study were used to correlate DNA adducts in the amphipods and the sediment contaminants, but a causative link remains to be established in future studies, for instance, by employing laboratory exposure experiments. Another concern is the insufficient characterization of the contaminants in the test sediments, as only PAH and metal data were available for our study. It is clear that the actual contaminant composition in these sediments was likely to be much more diverse.^73^ Unaccounted effects of other contaminants and, possibly, confounding effects on the DNA adduct profiles add uncertainty to our two-group classification of the sites based on only a fraction of the contaminants and not considering other chemical and nonchemical factors, such as temperature and oxygen conditions. Therefore, future studies must validate these findings to identify specific adducts associated with different exposures and nonchemical conditions that can modify the responses. Nevertheless, a successful separation (Figure 5) with an acceptable misclassification rate (25%) based on the ROC analysis suggests a high potential for this approach for screening DNA adduct profiles as a part of the effect-based assessment. Finally, measuring the contaminant body burden in the amphipods can provide further insights into the exposure levels and their association with DNA adducts.

There is a general agreement that current monitoring and assessment methods based on chemical concentrations in the environmental matrices (water, sediment, biota) are insufficient for protecting wild populations.^1^ DNA adductomics has emerged as a tool to detect oncogenic markers that can be used to identify cancers and other adverse effects in humans and wildlife^74^ and epigenetic marks to detect various disorders, such as inflammation, metabolic diseases, and developmental aberrations.^25,53,75,76^ This approach is applicable for the biological effect screening and monitoring in various species because only a small amount of the sample material is sufficient for the DNA adductome analysis. Our workflow for DNA adductomics has the advantage of screening and processing data for not only the low-mass adducts (e.g., epigenetics or oxidative adducts) but also bulky adducts, broadening the spectra of adducts detection that can be used for the environmental assessment. It is crucial, however, to focus on the structural identification of the modifications to improve the characterization of the exposure. Thus, including DNA adductomics in the existing batteries of biomarkers would provide a much-needed complement for early detection of the DNA-level alterations in wildlife and represent significant progress in the environmental health risk assessment.

## Data Availability

Data will be deposited to an open-access repository once the manuscript is accepted for publication.

## Supporting Information Available

Concentration of contaminants, statistical analysis (Pearson correlation, SIMPER, VIP score, ROC curves), adductome map, and chromatograms; environmental conditions, statistical analysis output (GLM, PERMANOVA, and unpaired *t*-test), and HPLC conditions (PDF)
Concentration of contaminants (XLSX)
DNA modification list (XLSX)
SIMPER analysis (XLSX)
