# Supplementary material for: DNA Adductomics for the Biological Effect Assessment of Contaminant Exposure in Marine Sediments
Source: Environ Sci Technol. 2023 Jun 21;57(29):10591–603. doi: 10.1021/acs.est.3c00499 (PMC10373492; doi:10.1021/acs.est.3c00499)
Supplement: Supplementary file 1 — es3c00499_si_001.pdf [file es3c00499_si_001.pdf]

Supplementary material

DNA adductomics for biological effect assessment of contaminant exposure in marine sediments

Giulia Martella[1], Elena Gorokhova[1], Pedro F.M.Sousa[2], Natalia Y. Tretyakova[3], Brita Sundelin[1] and Hitesh V. Motwani[1,*]

[1]Department of Environmental Science, Stockholm University, SE-106 91 Stockholm, Sweden

[2]Department of Materials and Environmental Chemistry, Stockholm University, SE-106 91 Stockholm, Sweden

[3]Department of Medicinal Chemistry and Masonic Cancer Center, University of Minnesota, Minneapolis, MN, 55455, United States

[*]Corresponding author: hitesh.motwani@aces.su.se

Table of Contents

CONTENT	Page
LIST OF NOTES	
**Note S1.** Chemical evaluation of contamination level in the sampled sites.	S3
**Note S2.** Optimization of LC-HRMS for adduct detection.	S6
LIST OF FIGURES	
**Fig. S1.** Classification results based on the total PAH concentration (SumPAH; µg/kg dwt) and Tomlinson Pollution Load Index (PLI) for metals in the Bothnian Sea (BS) and the Northern Baltic Proper (NBP).	S7
**Fig. S2.** Concentrations of LmPAHs and HmPAHs (µg/kg dwt) and total metal concentrations (Sum Metals; mg/kg dwt) in the Bothnian Sea (BS) and the Northern Baltic Proper (NBP) sites that were classified as Reference and Contaminated.	S8
**Fig. S3.** PCO biplot of reference (open symbols) and contaminated (filled symbols) stations in each basin.	S9
**Fig. S4.** Contribution of specific contaminants to the dissimilarity between the contaminated and reference sites according to SIMPER analysis based on the Euclidean matrices of the normalized data (left Y-axis).	S10
**Fig. S5.** Adductome map of detected adducts in four amphipod samples.	S11
**Fig. S6.** Chromatogram of BPDE-dG (100 fmol/µL) standard solution analyzed with HPLC method 3 (A) and method 5 (B) (Table S4), using ACN and MeOH as organic phase, respectively.	S12
**Fig. S7**. The heatmap of Pearson correlation among the adducts.	S13
**Fig. S8.** The heatmap of significant Pearson correlations between the relative adduct abundance and contaminants.	S14
**Fig. S9.** Pearson correlation coefficients for the adducts (low-mass: green; high-mass: red) and PAH concentrations for 11 congeners.	S15
**Fig. S10.** VIP scores of 15 top features of OPLS-DA for Component 1 (left) and validation of OPLS-DA by permutation test (Q^2: 0.4 and R^2Y: 0.855; right).	S16

**Fig. S11**. Area under the receiver operating characteristic (ROC) curve (AUC) using individual adducts identified as predictors in the OPLS-DA model.	S17
**Fig. S12**. Classification of the contamination status based on the three adducts (H483, L114 and L189) in the amphipod adductome. The classification was established using the area under the receiver operating characteristic (ROC) curve (AUC).	S18
LIST OF TABLES	
**Table S1.** Summary of environmental conditions in the study areas, the Bothnian Sea and Northern Baltic Proper, during the sampling period (January 2018).	S19
**Table S2.** Studies that provided contaminant concentration data to support the classification of the study sites as moderately-to-heavily contaminated and reference sites (low contamination levels).	S20
**Table S3.** Generalized Linear Model output.	S21
**Table S4.** PERMANOVA output for evaluating effects of basin (*Basin*) and contamination status (*Status*) on the concentrations of 10 metals and 11 PAH congeners in sediments.	S22
**Table S5.** HPLC methods tested for high mass adducts using BPDE-dG.	S23
**Table S6.** PERMANOVA output evaluating effects of basin (*Basin*) and contamination status (*Status*) on the DNA adduct responses.	S24
**Table S7.** Unpaired t-test comparing adduct-PAH correlation coefficients between the high- and low-mass adducts.	S25
**Table S8.** Unpaired t-test for the adducts found to be significantly upregulated or downregulated in the contaminated sites compared to the reference sites.	S26
REFERENCES	S27

LIST OF SUPPLEMENTARY FILES UPLOADED IN CSV FORMAT

CSV file S1. Concentrations of contaminants measured in the sediments as a part of Effect Screening Study in the Bothnian Sea (BS) and the Northern Baltic Proper (NBP). The 11 PAHs included phenanthrene (Phe), anthracene (Ant), fluorene (Flu), pyrene (Pyr), Benzo(a)anthracene (BaA), Chrysene (Chr), benzo[b]fluoranthene (BbF), benzo[k]fluoranthene (BkF), Benzo[a]pyrene (BaP), benzo[g,h,i]perlene (BghiP), and indeno [1,2,3-cd]pyrene (Ind). The results were expressed as a concentration in $ng \cdot g^{-1}$ dwt. The results for the metals (As, Cd, Co, Cr, Cu, Ni, Pb, Zn, and Hg) were expressed as a concentration in $mg \cdot kg^{-1}$ dwt. The values obtained in the table were calculated as explained in Note S1 of this supplementary material.

CSV file S2. List of the 119 adducts obtained from *nLossFinder* and used for data processing. The table includes the adduct name, the *m/z* observed of the precursor ions (representing nucleoside adducts; MS1) and of corresponding fragment ions (nucleobase adducts; MS2), the retention time (min) under the employed LC conditions, the absolute intensity and the peak areas for the MS1 and MS2 ions. The DIA window and the neutral loss error expressed as Δppm between the neutral loss 116.0473 and the observed value are also indicated.

CSV file S3. SIMPER outcome for selecting features responsible for 80% of the dissimilarities in the DNA adducts between the contaminated and reference sites. The average values for each adduct in the samples classified as *Contaminated* and *Reference*, the average squared distance, the ratio of the average contribution divided by the standard deviation (SD) of those contributions across all pairs of samples, and the relative contribution and the cumulative percentages are shown.

Note S1. Chemical evaluation of contamination level in the sampled sediments

Sampling

Sediment samples were collected on three occasions: (1) January 2018, as a part of the amphipod collection campaign in BS and NBP coordinated by the Effect Screening Study and SNMMP; (2) January 2012, as a part of the sediment contamination survey within SNMMP in BS and NBP; and (3) January 2011, within the recipient control study in BS (Table 1). The environmental conditions during sampling in the study areas are shown in **Table S1**. We selected 19 sites with well-characterized sediment contamination status, 9 in BS and 10 in the NBP (**Fig. 1**).

We used a benthic sled set for all field collections to sample the upper 2-3 cm of the sediment; thus, the measured concentrations represent the conditions in the uppermost sediment layer where the amphipods reside. The macrofauna was removed by sieving with 0.5-mm mesh, and the sediment was frozen at -20 °C.[1]

Chemical analyses

Aliquots of 20 g (wet mass) of each sediment sample was used to determine TOC values after drying and homogenization. We used a Flash 2000 (Thermo Scientific) elemental analyser at Stockholm University, Department of Ecology, Environment and Plant Science, and expressed TOC values as % of the dry weight (dwt). The percent moisture in sediment was determined by calculating the weight lost during the drying procedure and the values reported.

Aliquots of 50 g of the frozen sediment were used for PAHs (11 priority congeners)[2,3] at the Swedish Environmental Research Institute (IVL), Sweden and 1 g for metal (As, Cd, Co, Cr, Cu, Hg, Ni, Pb, V and Zn) analyses at ALS Scandinavia, Sweden. Both laboratories have accreditation for their analytical services.

The 11 major PAHs identified by the IVL included phenanthrene (Phe), anthracene (Ant), fluorene (Flu), pyrene (Pyr), Benzo(a)anthracene (BaA), Chrysene (Chr), benzo[b]fluoranthene (BbF), benzo[k]fluoranthene (BkF), Benzo[a]pyrene (BaP), benzo[g,h,i]perlene (BghiP), and indeno [1,2,3-cd]pyrene (Ind). The extraction and analysis of the PAH samples were performed using appropriate GC-MS methods with electron ionization by IVL according to SS-EN ISO / IEC 17025: 2018, the accredited method for PAH analysis. Details on methods, detection limits, quality control and other information can be found on the company website (https://www.ivl.se/english/ivl/our-offer/our-services/analysis-of-soil-and-sediment.html). The results were expressed as a concentration in ng g -1 dwt.

For the metal analysis in sediment samples, an aliquot of approximately 1 g of homogenized wet sediment was dried at 105 °C to constant weight. The metals (As, Cd, Co, Cr, Cu, Ni, Pb, Zn, and Hg) were analyzed by ICP-SFMS, ICP-AES and AFS at ALS Scandinavia; all analytical methods were accredited (SWEDAC). Details on methods, detection limits, quality control and other information can be found on the company website (https://www.alsglobal.se/en/environment/trace-metal-analysis). The results were expressed as a concentration in mg kg -1 dwt.

Data analysis

Classification principles. The chemical contaminant data were used to classify sampling sites into two categories (1) reference sites, where contaminant levels are relatively low, and the sediments are likely non-toxic and (2) contaminated sites, with at least one contaminant being at the potentially harmful levels (**Table S2**). As PAH and metal concentration data were available for all sampling sites, these two contaminant groups were used in the classification.

For the total assessment of the metal contamination degree, we used the Tomlinson Pollution Load Index (PLI) calculated as a geometric average of the concentration factors[4] for the nine metals (As, Cd, Co, Cr, Cu, Hg, Ni, Pb and Zn):

$PLI = \sqrt[n]{CF_1 + CF_2 + \cdots CF_n}$, where

CF is the concentration factor for each metal calculated as:

$CF = {C_{metal}}/{C_{baseline}}$, where

C_{metal} is the measured concentration of the metal (mg/kg dwt) and $C_{baseline}$ is the regional reference value for this metal[2]; n is the number of the metals included in PLI calculations.

As a criterion for the PAH contamination threshold for DNA adduct formation, we used a reported value of 290 µg/kg dwt established by a hockey-stick regression model relating DNA adduct formation in fish to sediment PAH concentration;[5] in this study, a mixture similar to the PAH composition in the test sediments from the Baltic Sea was used. The model approach was chosen to facilitate our site classification and identify exposure levels at which detectable and biologically relevant increases in DNA damage would be expected to occur in the amphipods. The alternative approach based on hazard quotients for individual PAHs and an overall hazard index was considered suboptimal because it would provide an estimate of the worst-case hazard of the sediment PAH to benthic organisms.[6] However, the latter would be toxicologically inconsistent with the hypothesis that this type of DNA damage is a precursor to more overt pathological conditions.

The site was classified as contaminated when either the PLI value exceeded 1 (i.e., due to the heavy metal pollution) or the sum of the PAH congeners (11 congeners measured in all sediments) exceeded 290 µg/kg dwt (due to PAH pollution). As the number of the PAH congeners was relatively low, our total PAH levels are likely underestimated, and, thus, some of the sites assigned as reference could have low-to-moderate PAH levels in sediments.

Statistical evaluation. To compare contaminant levels for LmPAHs and HmPAHs, and PLI between the basins, we used Generalized Linear Models with exponential distribution and reciprocal link as implemented in JMP (version 15). Moreover, the complete datasets for PAH and metal concentrations were analyzed by main and pairwise permutational analysis of variance (PERMANOVA) in PRIMER v.6.4[7] with 999 permutations at a significance level of $p<0.05$. The concentrations were log(x+1) transformed and used to calculate Euclidean similarity matrices before the analysis. The homogeneity of dispersion test (PERMDISP) was carried out based on calculated distances to centroids to assess whether significant PERMANOVA results were based on location or dispersion effects. The PERMANOVA design included *basin* (BS and NBP) and *contamination status* (contaminated and reference sites) as factors; the interaction was also considered for testing whether the contaminants contributed differently in different basins. The model outcome was visualized by PCO plots. Finally, a similarity percentage (SIMPER) analysis was run to determine contaminant categories contributing most to the differences between the reference and contaminated sites.

Contaminant assessment

The contaminant levels were variable across the sites, and hot spots of contamination were identified for PAHs and metals (Cu, Zn, Pb, As, Cr and Hg). Moreover, some locations exceeded acceptable values provided by the sediment quality guidelines. Thus, a high level of contamination in some areas and high variability in the contaminants were observed even at a small spatial scale (**Fig. S1**).

Using total PAH concentrations and PLI values based on the metal concentration for all 19 sites, we classified them into reference (3 and 6 for BS and NBP, respectively) and contaminated (5 in each basin) sites (**Fig. 1** and **Table 1**). Notably, stations used as relatively unpolluted in the SNMMP (6020, 6022, and 6025) were classified as contaminated due to both PLI and total PAH levels exceeding the threshold values.

Using total concentrations of the low- (LmPAHs) and high-mass PAHs (HmPAHs) and metals, we found no significant *basin* × *contaminant status* interaction and no significant *basin* effect on the contaminant

levels (**Table S3**). There was high variability in the contaminant levels between the sites as indicated by the Principle Coordinate Analysis (PCO; **Fig. S3**) following PERMANOVA that identified *Contamination status* effect as the only significant factor. The results of PERMDISP showed that the dispersion was not significantly different between the groups (**Table S4**); thus, the observed effect was due to the distance between the centroids and their location. The ordination biplot (**Fig. S3**) confirmed that metal contamination was mainly associated with US5 and, to some extent, SU4 sites, whereas high levels of hydrocarbons were associated with the sites 57-58 and Norrsundet. Thus, the difference between the sites assigned as *Contaminated* and *Reference* was significant and not affected by *Basin*. Moreover, all contaminant groups (i.e., metals, LmPAHs, and HmPAHs) were significantly higher in the sediments classified as contaminated (**Fig. S2**).

The metals were primarily responsible for 80% of the dissimilarity between the contaminated and reference sites as suggested by SIMPER analysis following the PERMANOVA results (**Table S4** and **Fig. S4**), with the most influential metals being Cu, Zn and Pb. A set of PAHs contributing to the dissimilarity between the site categories included both HmPAHs (BKF, BAA and ICDP) and LmPAHs (PA, PYR, BBF, and FLU), each contributing about 3.5%.

Note S2. Optimization of LC-HRMS for adduct detection.

Two LC-HRMS methods were used for detecting the nucleoside adducts, one for low-mass adducts (up to *m/z* 350), and the other for high-mass adducts (350-600 *m/z*). The method for low-mass adducts was presented in our earlier work.[8] For high-mass adducts, optimization of the analytical methodology was performed using BPDE-dG (100 fmol/µL) as a standard.

The optimization was based on having a suitable chromatographic elution and detecting the adduct (if unknown) by the FS/DIA approach. Five HPLC gradients were tested on the Ascentis Express F5 column (**Table S5**). Also, two mobile phase systems were tested; water and acetonitrile (ACN) with 0.1% formic acid and water and methanol (MeOH) with 0.1% formic acid. The MS detection method included FS coupled with DIA with an inclusion list for high-mass range adducts 350-600 *m/z*.

Concerning chromatography, it was important that BPDE-dG, a hydrophobic adduct, eluted late in the program to allow for relatively less hydrophobic adducts that eluted earlier to be separated. Several elution methods were tested (**Table S5)**. Two of the HPLC methods (methods 1 and 2; **Table S5**) tested with ACN as an organic phase resulted in a late elution of BPDE-dG at the washing step, where the percentage of B was at its maximum. At this point, BPDE-dG elutes with probable matrix impurities in the column, which can result in matrix effects such as ion suppression, decreasing the sensitivity and, thus, the response of the analyte. Another method used with ACN included a 28 min gradient (method 3; **Table S5**), where BPDE-dG eluted at 13.67 min, and the washing step started at 20 min. When employing HPLC method 5 (**Table S5**) with MeOH as the mobile phase, the analyte eluted at 20 min while the washing step of the program started at 22 min (**Fig. S6**). As MeOH was used as the organic phase, the analyte eluted later due to the lower eluotropic strength of MeOH compared to ACN. Although the analyte eluted later in the gradient, it still occurred before the washing step. Moreover, as the same mobile phase with MeOH was used for the LC-MS analysis of the low-mass adducts, this method was selected as an optimal HPLC method for the high-mass nucleoside adducts.

BPDE-dG has a protonated molecular ion, $[M+H]^+$, with *m/z* 570.1989. The specific fragment ion of BPDE-dG, i.e., the nucleobase-adduct, obtained by the neutral loss of deoxyribose gives an *m/z* 454.1511. Therefore, this fragment ion should be present in the MS2 spectrum and the molecular ion in MS1 spectrum for BPDE-dG to be selected by the *nLossFinder* program as a possible adduct. Both ions were present in their respective spectrum with relatively high intensities and high mass accuracy (± 5 ppm; method 5, **Table S5**; **Fig. S6**); also seen were other characteristic fragments of BPDE-dG. Therefore, this method was selected for the screening of high-mass adducts.

Fig. S1. Classification results based on the total PAH concentration (SumPAH; µg/kg dwt) and Tomlinson Pollution Load Index (PLI, unitless**) for metals in the Bothnian Sea (BS) and the Northern Baltic Proper (NBP).** The SumPAH and PLI values are shown for the sites that were classified as reference (Status: 0) and contaminated (Status: 1). The horizontal lines indicate threshold values for SumPAH (equals 290 µg/kg dwt; blue) and PLI (equals 1; red). Sampling sites are listed on the X-axis.

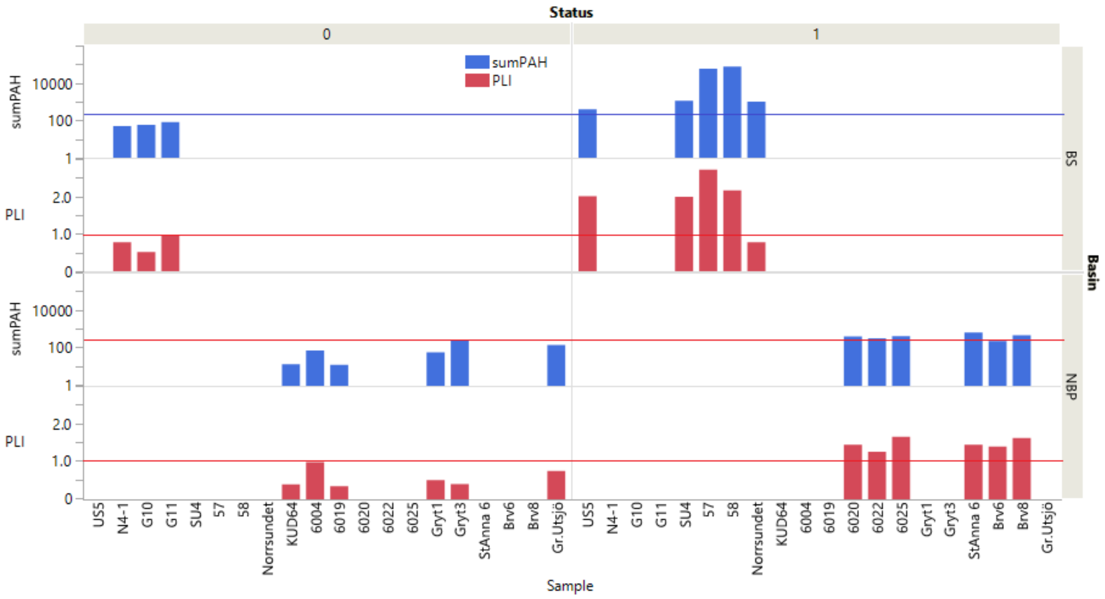

Fig. S2. Concentrations of LmPAHs and HmPAHs (µg/kg dwt) and total metal concentrations (Sum Metals; mg/kg dwt) in the Bothnian Sea (BS) and the Northern Baltic Proper (NBP) sites that were classified as Reference and Contaminated. The distribution of each dataset is shown: the maximum, minimum, median, and the range on either side of the median.

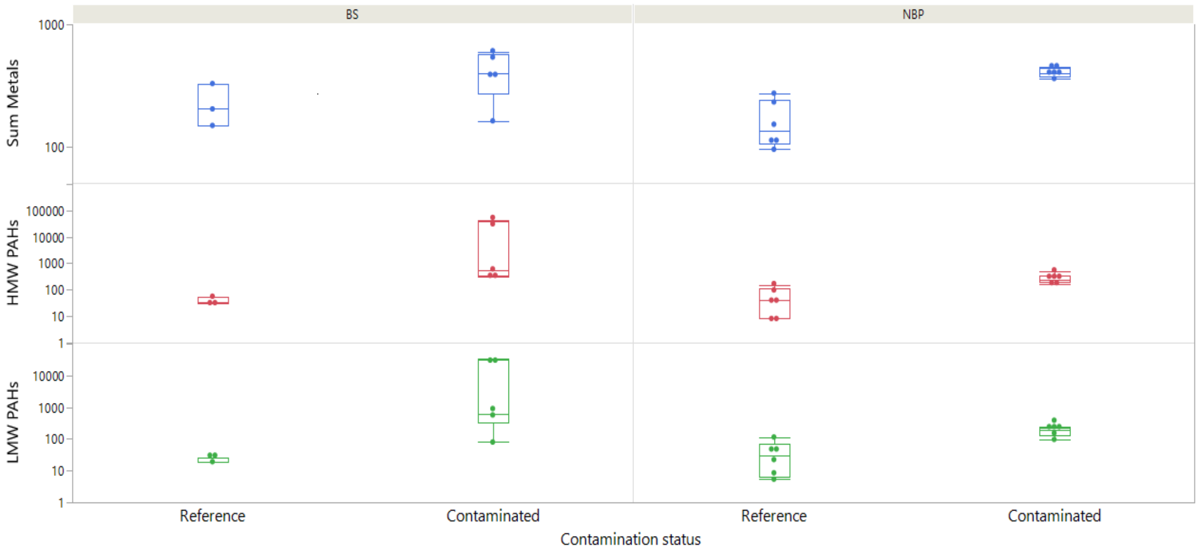

Fig. S3. PCO biplot of reference (open symbols; BS0 and NBP0) and contaminated (filled symbols; BS1 and NBP1) sites in each basin. The analysis was based on the Euclidean similarity matrix calculated from log-transformed concentration values for PAHs and metals. The sites are shown as blue (Bothnian Sea, BS) and red (Northern Baltic Proper, NBP) dots.

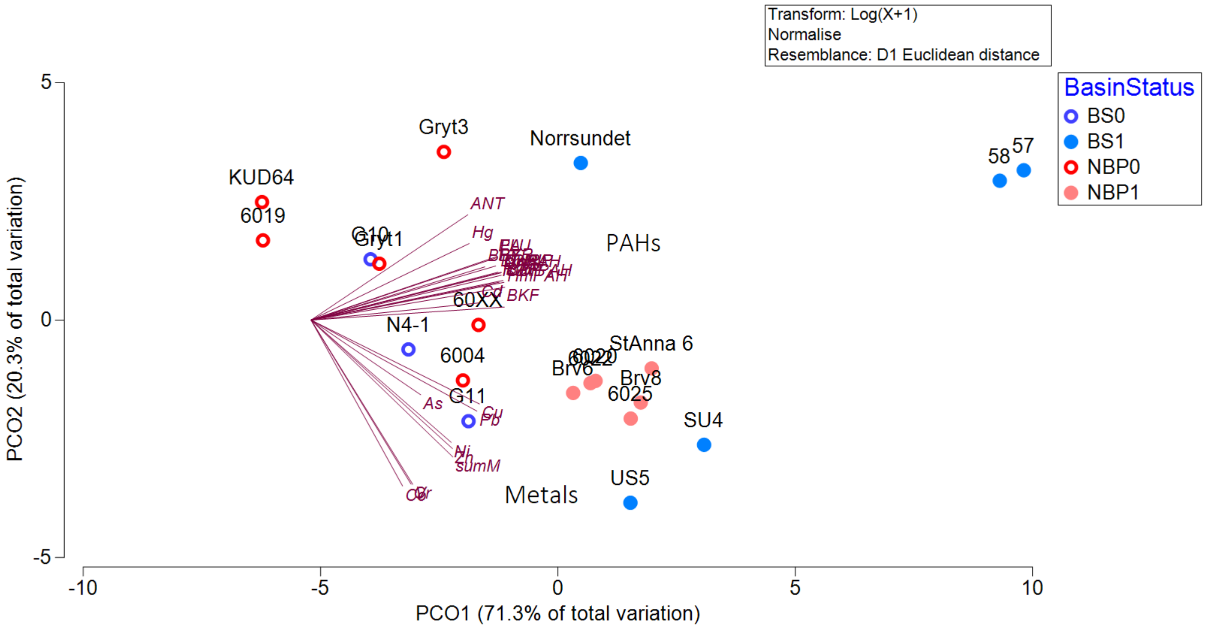

Fig. S4. Contribution of specific contaminants to the dissimilarity between the contaminated and reference sites according to SIMPER analysis based on the Euclidean matrices of the normalized data (left Y-axis). The cut-off was set to 80% of the cumulative percentage explained by the data (right Y-axis). The metals contributed most (3.6% – 9.9%) to the overall dissimilarity, followed by a set of HmPAHs (shown in bold) and LmPAHs (in Italics), with each congener contributing about 3.5%.

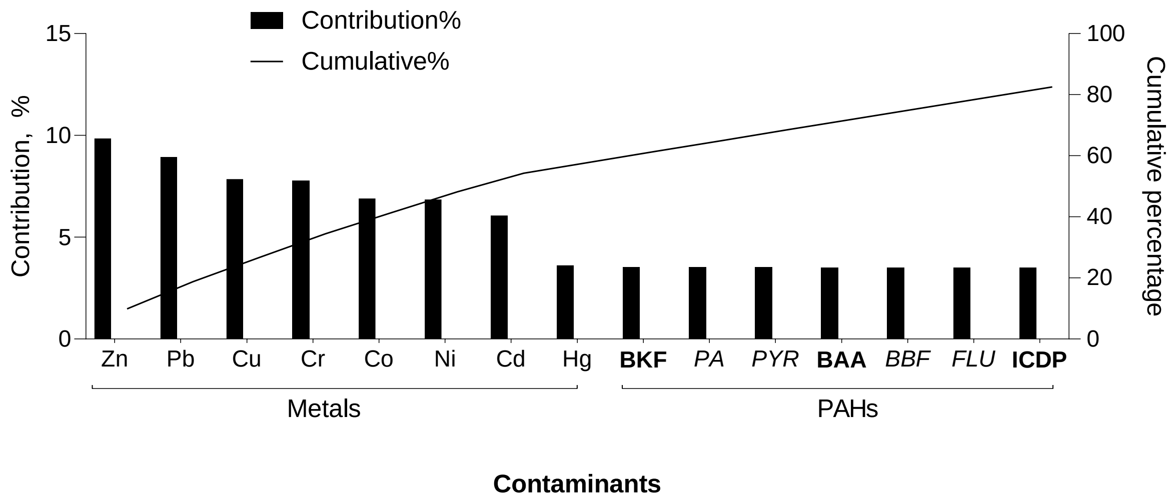

Fig. S5. Adductome map of detected adducts in four amphipod samples. Adduct profiles were obtained by nontargeted screening using *nLossFinder*. Each circle represents a nucleoside adduct. Retention time using the employed LC-condition is shown on the X-axis, *m/z* of detected precursor ion on the Y-axis, and the circle size corresponds to the adduct's peak area (normalized to dG, as peak area-adduct ×100/peak area-dG).

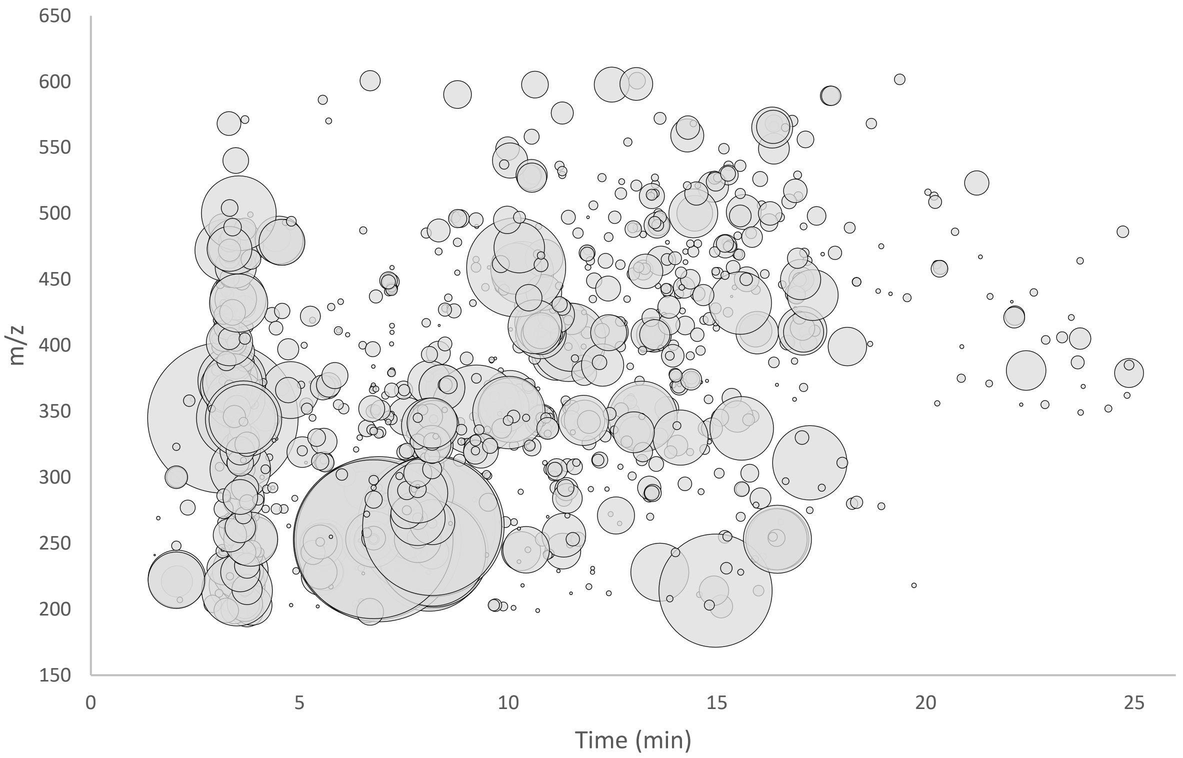

Fig. S6. Chromatogram of BPDE-dG (100 fmol/µL) standard solution analyzed with HPLC method 3 (A) and method 5 (B), using ACN and MeOH as organic phase, respectively (Table S5). Note the EIC of precursor ion (not observed with method 3) and the five fragments, including the nucleobase adduct fragment (*m/z* 454.1511), and the common fragment representing protonated deoxyribose (*m/z* 117.0545), with ±3 ppm mass accuracy. For structures of the fragments, see (Motwani 2020).[9]

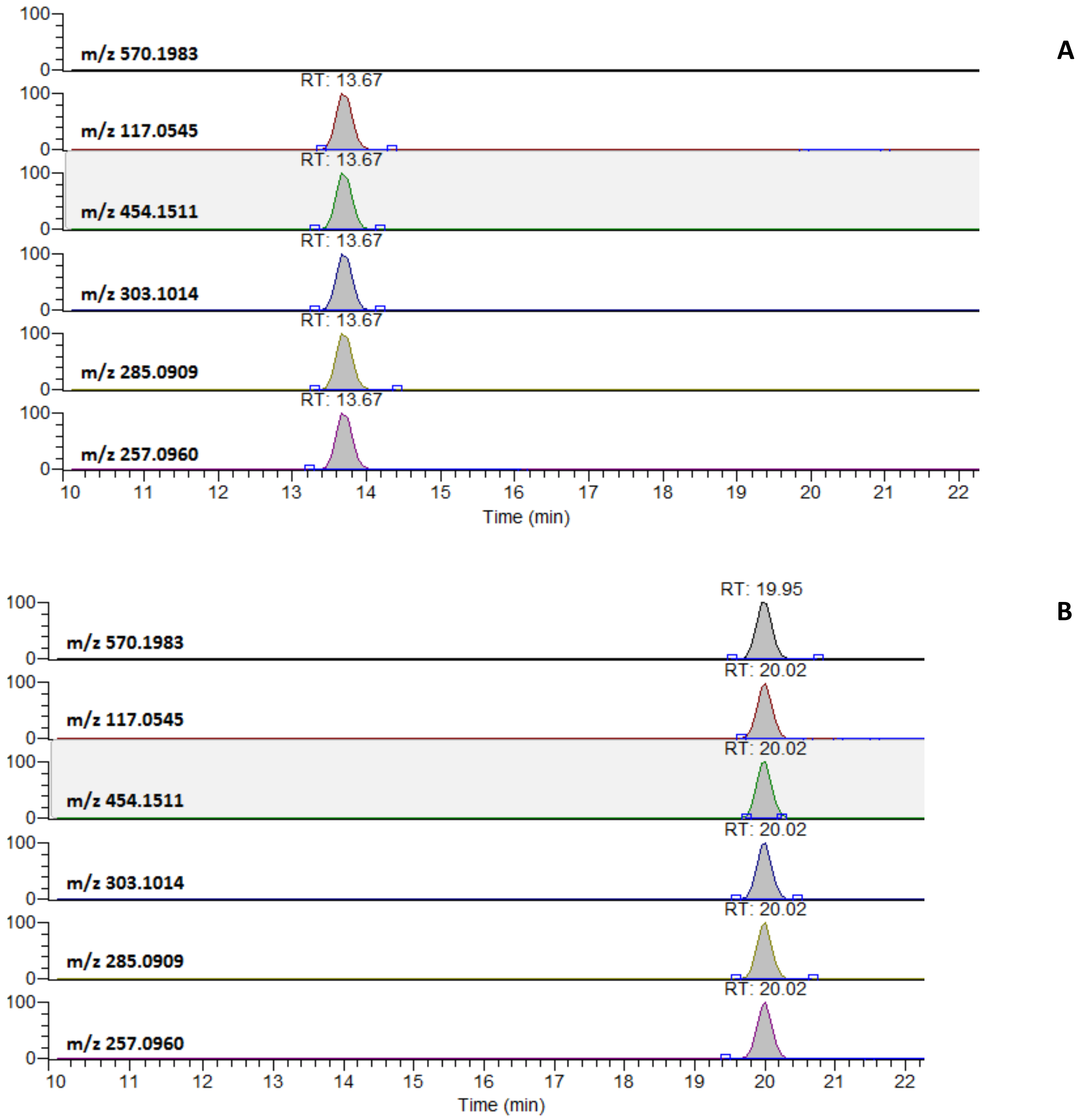

Fig. S7. **The heatmap of Pearson correlations among the adducts.** The colour shade in each square block indicates the strength and the direction of the correlation (positive or negative) between a pair of adducts. LMs, low-mass adducts; HMs, high-mass adducts. Note the lower frequency of the positive correlations across the LMs than the HMs, and even lower LM-HM correlations.

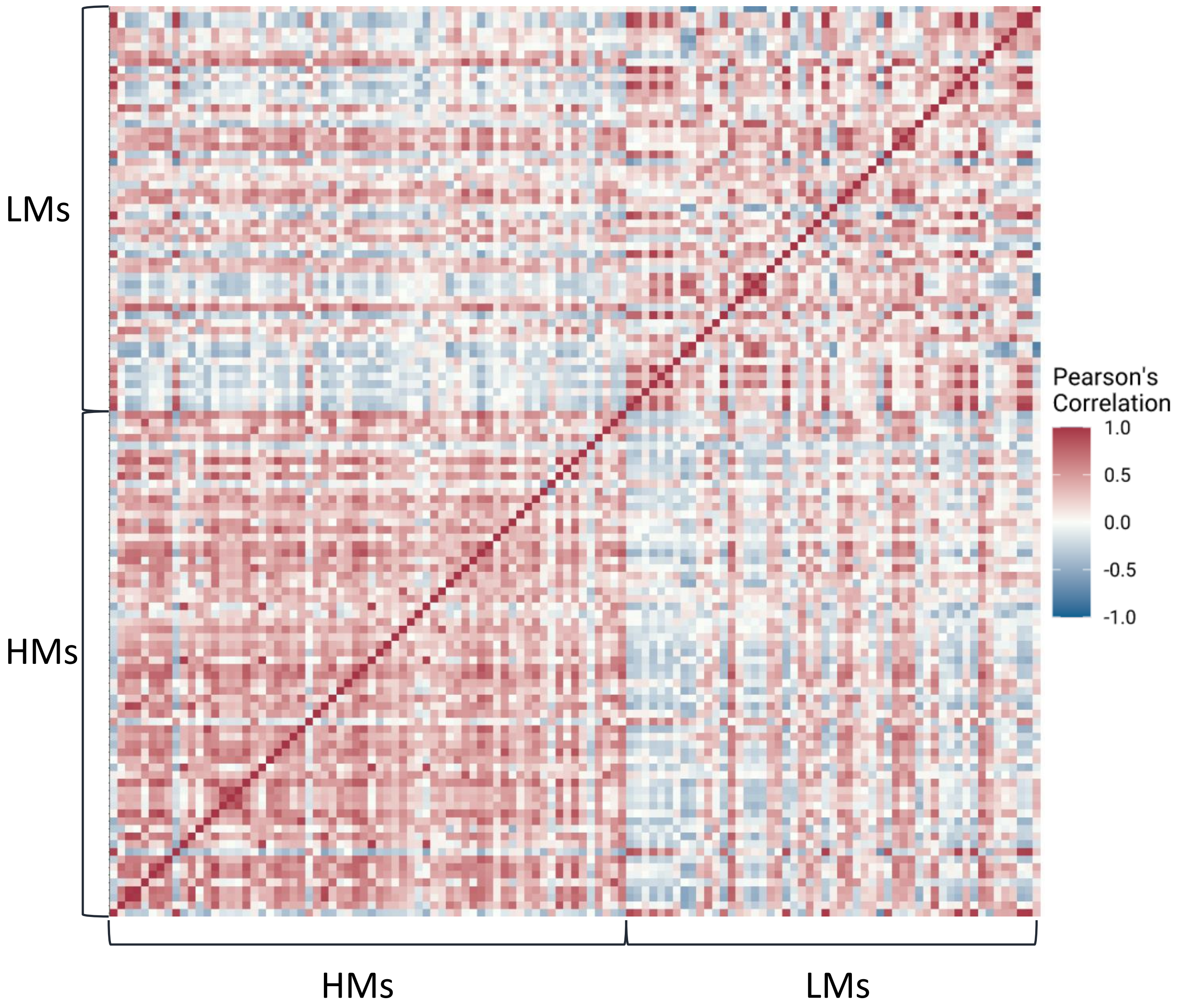

Fig. S8. The heatmap of significant Pearson correlations between the relative adduct abundance and the contaminants. (A) the low-mass adducts and (B) the high-mass adducts. Each dot is a significant ($p < 0.05$) correlation with colour and shade, indicating the correlation direction (positive in blue and negative in red) and its strength (Pearson *r*: -0.62 to 0.25), with more intense shading and bigger circle size for stronger correlations.

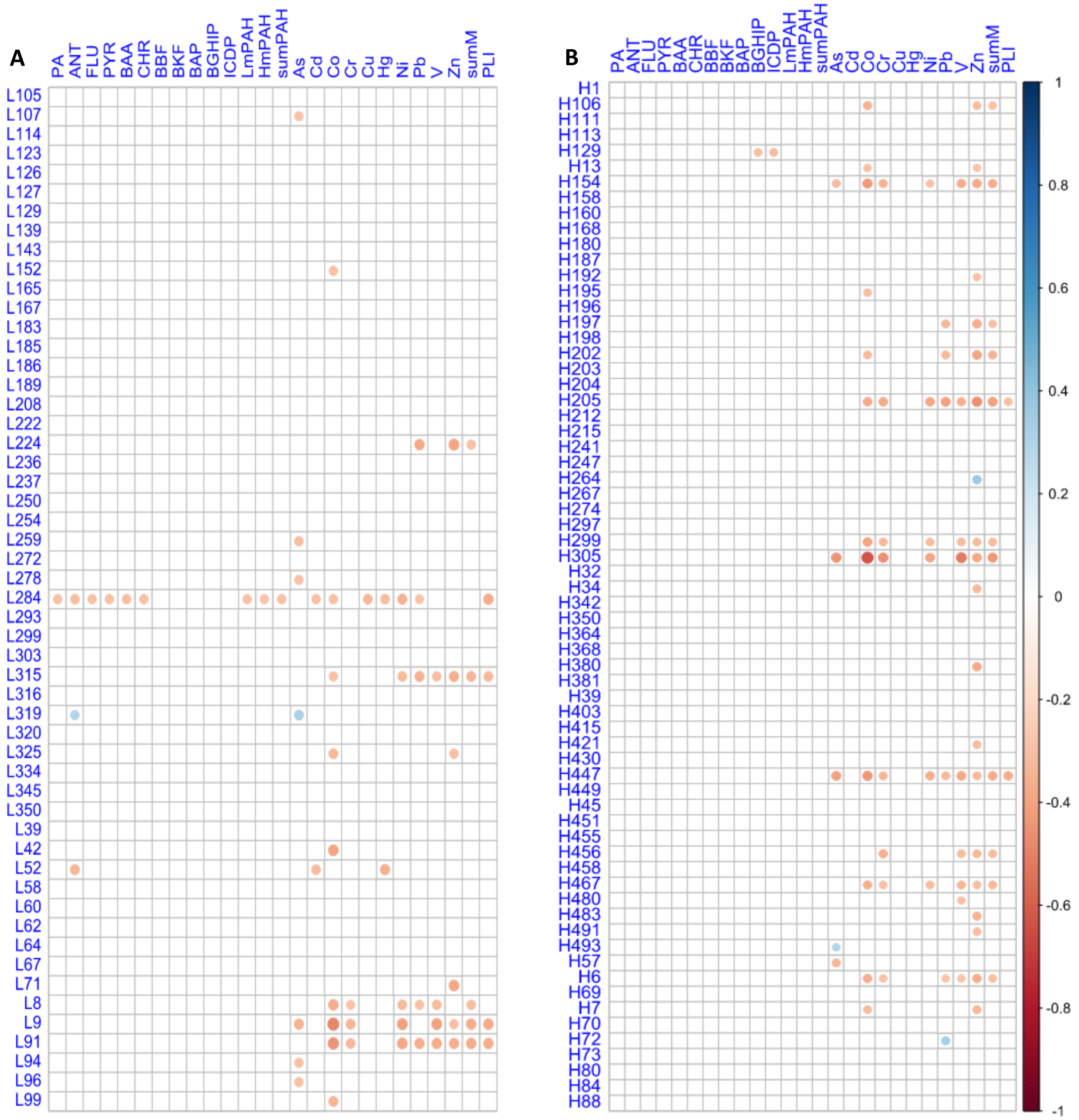

Fig. S9. Pearson correlation coefficients for the adducts (low-mass: green; high-mass: red) and PAH concentrations for 11 congeners. In all congeners, LmPAHs, HmPAHs and the total PAHs, the correlation coefficient values were higher for the high-mass adducts than the low-mass adducts. The box-and-whiskers graphs show the minimum and maximum values (line extending parallel to the box), the lower and upper quartile (extremes of the box), the median (black bar) and the mean (yellow dot).The red line shows the optimal cut-off.

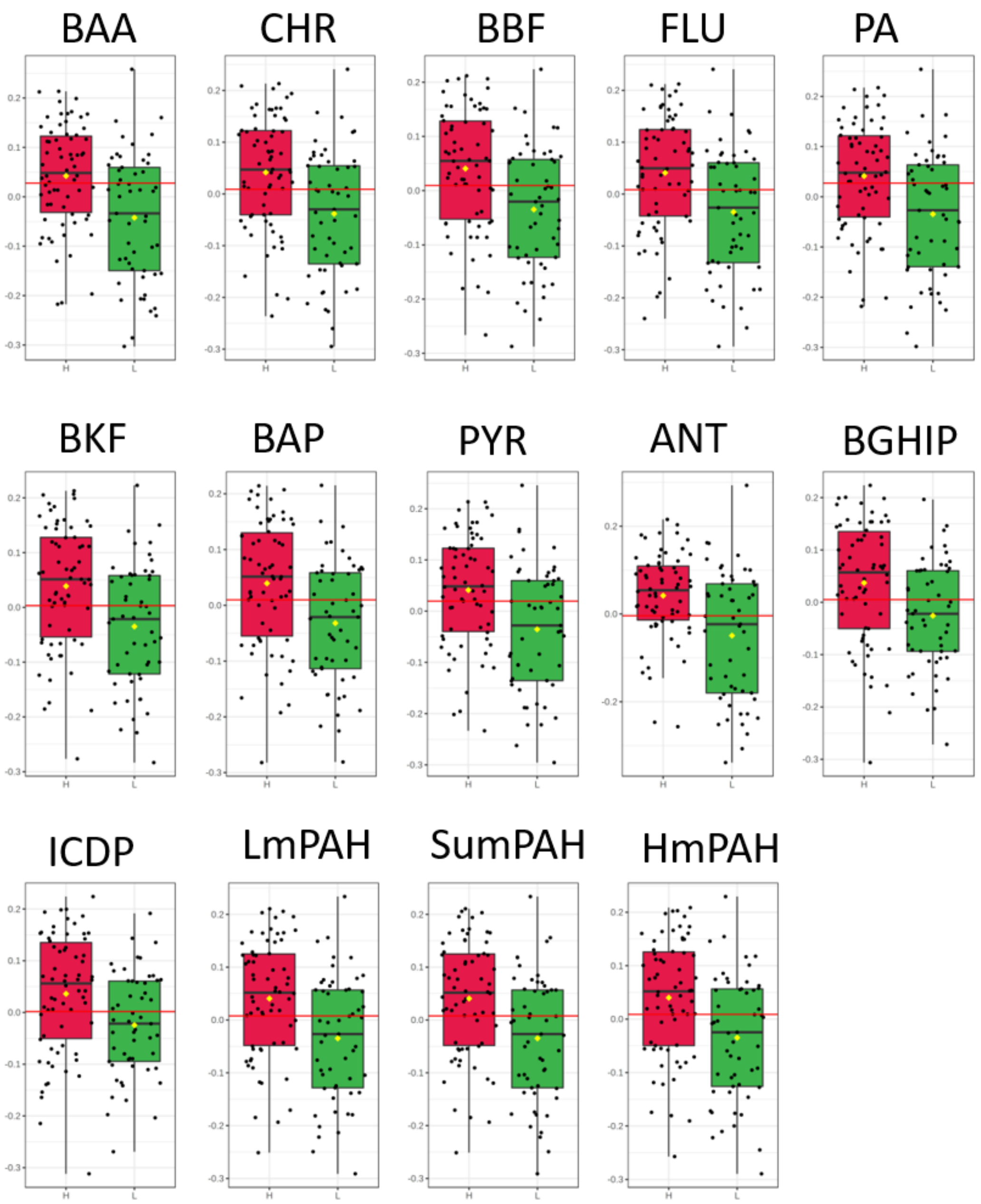

Fig. S10. VIP scores of 15 top features of OPLS-DA for Component 1 (left) and validation of OPLS-DA by permutation test (Q^2: 0.4 and R^2Y: 0.855; right).

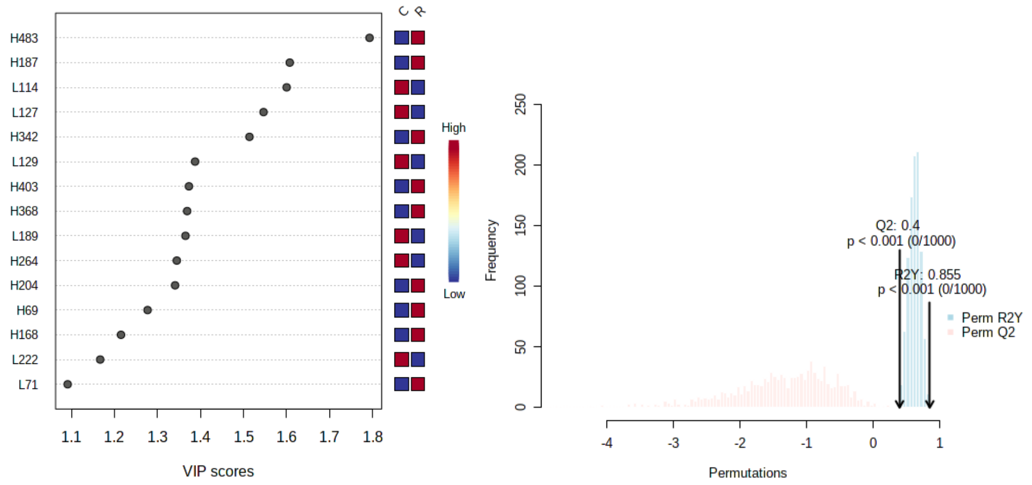

Fig. S11. **Area under the receiver operating characteristic (ROC) curve (AUC) using individual adducts identified as predictors in the OPLS-DA model.** The 45-degree diagonal represents the expected projection of a random classifier with no predictive value. The X-axis represents the false positive rate, while the Y-axis represents the true positive rate. For each of the 11 discriminating putative adducts (the adduct name is shown on top), the left panel is the AUC and the corresponding confidence interval, and on the right, the boxplot with the normalized values for the adducts in *Contaminated* (red) and *Reference* (green) stations, with the red line showing the optimal cut-off. The box-and-whiskers graphs show the minimum and maximum values (line extending parallel to the box), the lower and upper quartile (extremes of the box), the median (black bar) and the mean (yellow dot).

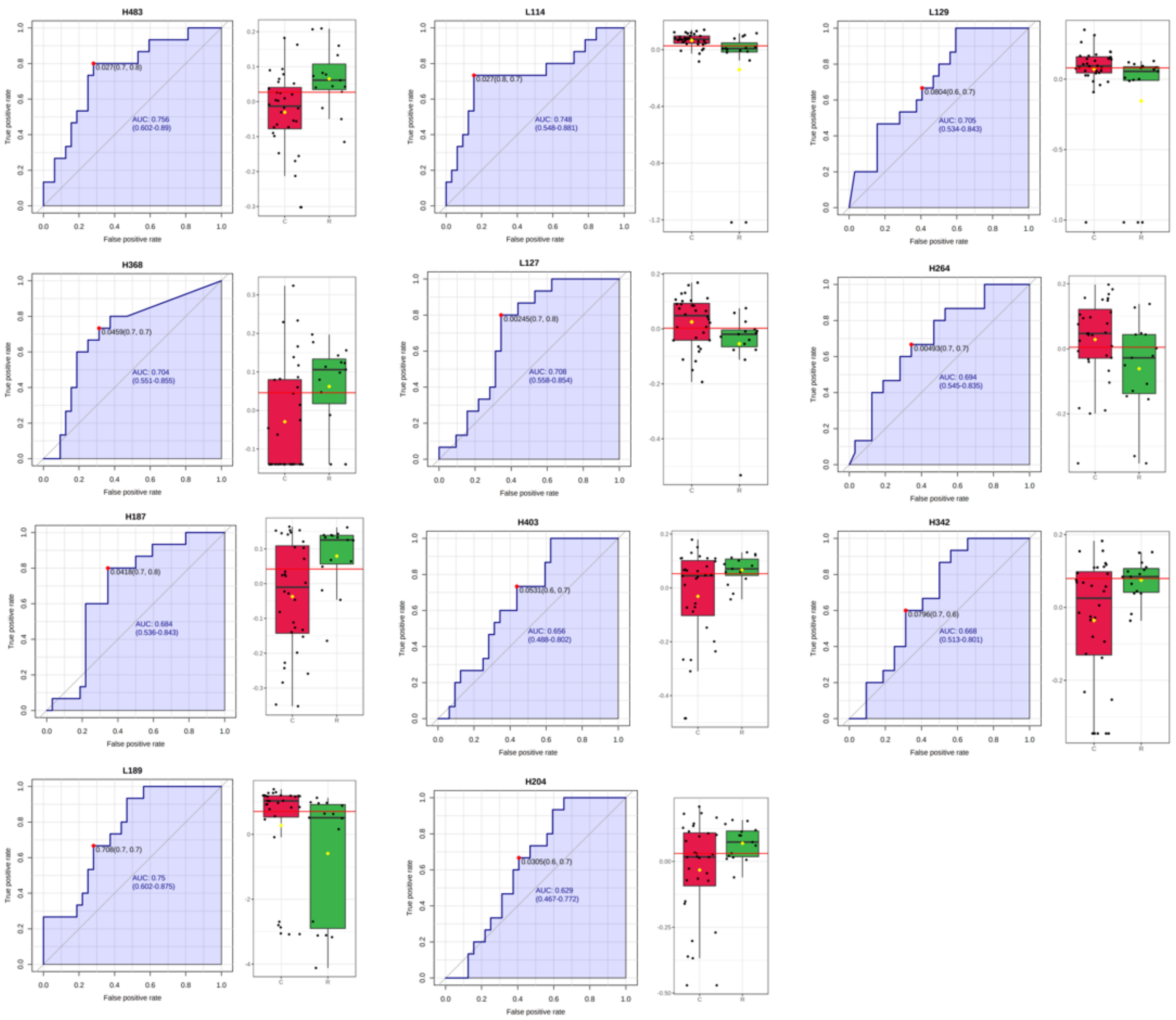

Fig. S12. **Classification of the contamination status based on the three adducts (H483, L114 and L189) in the amphipod adductome. The classification was established using the area under the receiver operating characteristic (ROC) curve (AUC).** The AUC with the optimal value of 0.825, the confidence interval, and the confusion matrix are shown.

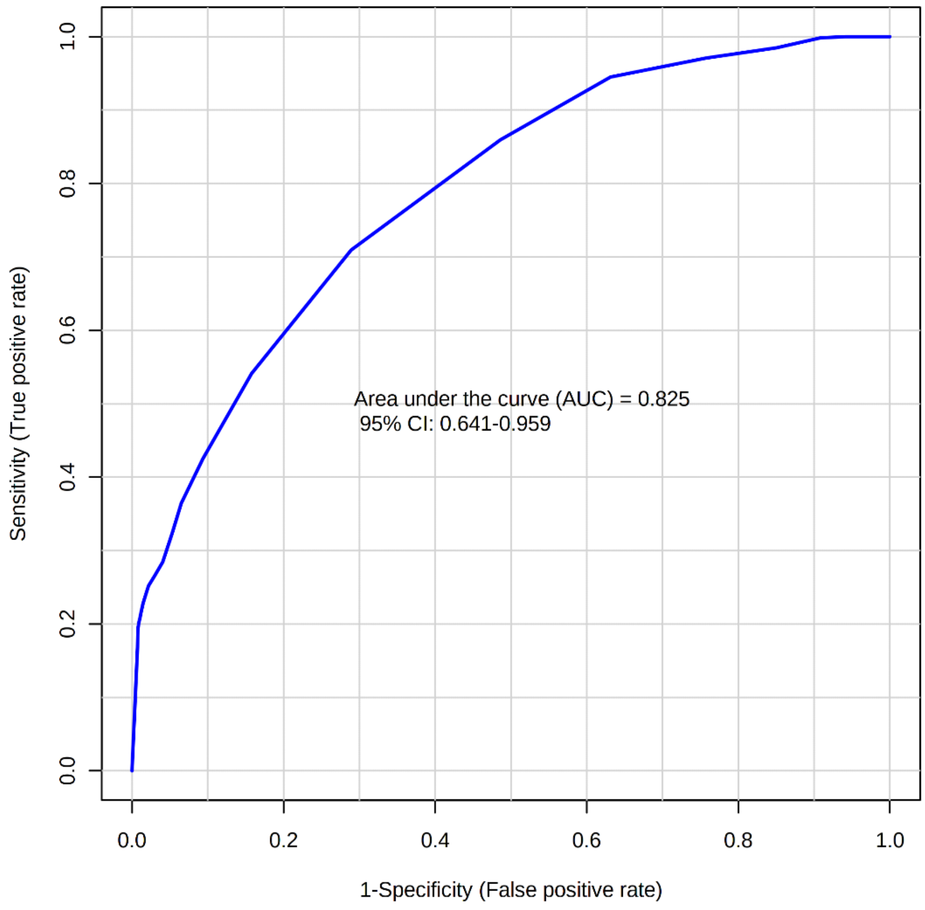

Confusion Matrix

	**C**	**R**
**C**	27	5
**R**	5	10

Table S1. Summary of environmental conditions in the study areas, the Bothnian Sea and Northern Baltic Proper, during the sampling period (January 2018). The mean values were calculated for all stations within a basin, and the range (in parentheses, min – max) shows the variability between the sampled stations. The temperature, salinity and oxygen data are for the bottom layer of the water column, and total organic carbon (TOC) values are for the uppermost layer of sediment.

**Environmental parameters**	**Bothnian Sea**	**Northern Baltic Proper**
Bottom depth, m	36 (12 – 137)	37 (13 – 77)
Temperature, °C	3.2 (<3 – 4.1)	3.8 (3.3 – 4.7)
Salinity	5.8 (5.6 – 6.3)	6.1 (5.9 – 6.9)
Oxygen, mg/L	8.2 (7.7 – 9.9)	7.8 (7.1 – 10.1)
TOC, %	1.2 (0.4 – 3.8)	2.1 (0.7 – 3.3)

Table S2. Studies that provided contaminant concentration data for classification of the study sites as moderately-to-heavily contaminated and reference sites (i.e., low contamination levels).

Campaign name	Stations	Contaminants analyzed	Reference
Effect Screening Study	57, 58, Norrsundet, Bråviken 5, 6004, 6019, 6020, Bråviken 6, and Bråviken 8.	PAHs, Dioxins, WHO-PCBs, OCPs, PBDEs, HBCDs, PCBs, PFAS, Organotin/TBT, Metals	[10]
SNMMP survey	N 4-1, G 10, G 11, KUD 64, Gryt 1, Gryt 3, and 6022.	PAHs, Metals	[11]
Recipient control survey in BS	US5 and SU4	PAHs, PCBs, Metals	[12]

Table S3. Generalized Linear Model output for evaluating effects of basin (*Basin;* BS: Bothnian Sea, NBP: Northern Baltic Proper) and contamination status (*Status;* 0: Reference, 1: Contaminated) on PLI (Pollution Load Index for metals), LmPAHs and HmPAHs (low- and high-molecular PAHs, respectively). Significant effects are indicated by colour; See Fig. S2 for the data visualization.

Term	Estimate	Std Error	L-R ChiSquare	Prob>ChiSq	Lower CL	Upper CL
**PLI**						
Basin[BS]	0.1558207	0.0830119	3.2453231	0.0716	-0.015014	0.3266554
Status[0]	-0.52232	0.0830119	21.835318	<.0001	-0.693154	-0.351485
Basin[BS]*Status[0]	-0.066214	0.0830119	0.6263214	0.4287	-0.237048	0.104621
**LmPAHs**						
Basin[BS]	0.0032731	0.0066882	0.2771612	0.5986	-0.007374	0.0208375
Status[0]	0.0154062	0.0066882	18.727134	<.0001	0.0057873	0.0330933
Basin[BS]*Status[0]	0.0059762	0.0066882	1.0410275	0.3076	-0.004663	0.0235421
**HmPAHs**						
Basin[BS]	0.0010055	0.0039946	0.0674924	0.7950	-0.005716	0.0112754
Status[0]	0.0095658	0.0039946	18.677922	<.0001	0.0036983	0.0199644
Basin[BS]*Status[0]	0.0027683	0.0039946	0.56794	0.4511	-0.003948	0.0130394

Table S4. PERMANOVA output evaluating effects of basin (*Basin*) and contamination status (*Status*) on the concentrations of 10 metals and 11 PAH congeners in sediments. The PERMDISP test results are: F = 2.767, $df_1 = 1$ $df_2 = 18$; P(perm) = 0.292.

Source	df	SS	MS	Pseudo-F	P(perm)
Basin	1	28.016	28.016	2.189	0.111
Status	1	191.14	191.14	14.937	0.001
Basin*Status	1	29.252	29.252	2.286	0.101
Res	16	204.74	12.796		
Total	19	475			

Table S5. HPLC methods tested for high mass adducts using BPDE-dG. Mobile phases employed were 95% water + 5% organic solvent with 0.1% formic acid (phase A), and 95% organic solvent +5% water with 0.1% formic acid (phase B). The organic solvent used was either methanol (MeOH) or acetonitrile (ACN). Injection volume was 10 μL.

Method number	Organic phase	Flow rate (ml/min)	Gradient program
1	ACN	0.125	25 min gradient. 5% B for 2 min, linear increase to 30% B at 9 min, then a linear increase to 100% B at 15 min, which was held for 5 min. Linear decrease back to 2% B in 1 min, held for 4 min.
2	ACN	0.125	23 min gradient. 5% B for 2 min, linear increase to 30% B at 9 min, then a linear increase to 100% B at 12.5 min which was held for 5 min. Back to initial conditions at 18 min, held for remaining 5 min.
3	ACN	0.125	28 min gradient. 5% B for 2 min, linear increase to 30% B at 9 min. Linear increase to 100% B at 20 min, held for 3 min. Back to initial conditions and held for 5 min.
4	MeOH	0.125	28 min gradient. 5% B for 2 min, linear increase to 30% B at 9 min. Linear increase to 100% B at 22 min, held for 4 min. Back to initial conditions and held for 2 min.
5	MeOH	0.125	30 min gradient. 5% B for 2 min, linear increase to 30% B at 9 min. Linear increase to 100% B at 22 min, held for 6 min, then back to initial conditions, held for 2 min.

Table S6. PERMANOVA output evaluating effects of basin (*Basin*) and contamination status (*Status*) on the DNA adduct responses. The PERMDISP test results are: F = 0.0089; P(perm) = 0.9.

**Source**	**df**	**SS**	**R2**	**Pseudo-F**	**P(perm)**
Status	1	4.912	0.04335	2.1018	0.019
Basin	1	4.557	0.04022	1.9501	0.025
Status*Basin	1	3.340	0.02948	1.4294	0.131
Res	43	100.484	0.88694		
Total	46	113.293	1.00000		

Table S7. Unpaired t-test comparing adduct-PAH correlation coefficients between the high- and low-mass adducts. The significant PAHs are in bold ($p < 0.05$). In all cases, the high-mass adducts had higher correlations than the low-mass adducts; see Fig. S9.

PAHs	T statistics	p value	-log10(p)	False Discovery Rate (FDR)
ANT	4.0124	**0.0001**	3.9733	0.0007
BAA	3.9318	**0.0001**	3.8438	0.0007
CHR	3.7797	**0.0002**	3.6043	0.0007
BBF	3.6210	**0.0004**	3.3614	0.0007
PYR	3.6139	**0.0004**	3.3508	0.0007
LmPAH	3.6035	**0.0004**	3.3351	0.0007
sumPAH	3.5996	**0.0004**	3.3291	0.0007
HmPAH	3.5946	**0.0004**	3.3217	0.0007
FLU	3.5875	**0.0004**	3.3111	0.0007
BKF	3.5488	**0.0005**	3.2532	0.0007
PA	3.5461	**0.0005**	3.2493	0.0007
BAP	3.4574	**0.0007**	3.1185	0.0008
BGHIP	3.0982	**0.0024**	2.6129	0.0026
ICDP	3.0109	**0.0031**	2.4960	0.0031

Table S8. Unpaired t-test for the adducts found to be significantly upregulated or downregulated in the contaminated sites compared to the reference sites. The significant PAHs are in bold ($p < 0.05$).

Adduct	T statistics	p.value	-log10 (p)	False Discovery Rate (FDR)
H483	-2.8339	**0.0068**	2.1636	0.1459
L114	2.6529	**0.0109**	1.9592	0.1459
H187	-2.6017	**0.0125**	1.9027	0.1459
H342	-2.4215	**0.0195**	1.7089	0.1526
L129	2.3433	**0.0235**	1.6272	0.1526
L127	2.2997	**0.0261**	1.5824	0.1526
H368	-2.1958	**0.0333**	1.4775	0.1558
H264	2.1470	**0.0372**	1.4293	0.1558
H403	-2.0691	**0.0443**	1.3535	0.1558

References

(1) Löf, M.; Sundelin, B.; Liewenborg, B.; Bandh, C.; Broeg, K.; Schatz, S.; Gorokhova, E. Biomarker-Enhanced Assessment of Reproductive Disorders in Monoporeia Affinis Exposed to Contaminated Sediment in the Baltic Sea. *Ecological Indicators* **2016**, *C* (63), 187–195. https://doi.org/10.1016/j.ecolind.2015.11.024.

(2) SEPA. *Bedömningsgrunder För Miljökvalitet. Kust Och Hav*; Naturvårdsverket rapport 4914; SEPA: Uppsala, 1999.

(3) Josefsson, S. *Klassning av halter av organiska föroreningar i sediment*; 2017:12; Uppsala, 2017; p 14.

(4) Tomlinson, D.; Wilson, J.; Harris, C.; Jeffrey, D. Problems in the Assessment of Heavy-Metal Levels in Estuaries and the Formation of a Pollution Index. *Helgol. Meeresunters.* **1980**, *33*, 566–575.

(5) Johnson, L. L.; Collier, T. K.; Stein, J. E. An Analysis in Support of Sediment Quality Thresholds for Polycyclic Aromatic Hydrocarbons (PAHs) to Protect Estuarine Fish. *Aquatic Conservation: Marine and Freshwater Ecosystems* **2002**, *12* (5), 517–538. https://doi.org/10.1002/aqc.522.

(6) Neff, J. M.; Stout, S. A.; Gunster, D. G. Ecological Risk Assessment of Polycyclic Aromatic Hydrocarbons in Sediments: Identifying Sources and Ecological Hazard. *Integr Environ Assess Manag* **2005**, *1* (1), 22. https://doi.org/10.1897/IEAM_2004a-016.1.

(7) Anderson, M. J. A New Method for Non-Parametric Multivariate Analysis of Variance: NON-PARAMETRIC MANOVA FOR ECOLOGY. *Austral Ecology* **2001**, *26* (1), 32–46. https://doi.org/10.1111/j.1442-9993.2001.01070.pp.x.

(8) Gorokhova, E.; Martella, G.; Motwani, N. H.; Tretyakova, N. Y.; Sundelin, B.; Motwani, H. V. DNA Epigenetic Marks Are Linked to Embryo Aberrations in Amphipods. *Scientific Reports* **2020**, *10* (1), 655. https://doi.org/10.1038/s41598-020-57465-1.

(9) Motwani, H. V. DNA as an in Vitro Trapping Agent for Detection of Bulky Genotoxic Metabolites. *Journal of Chromatography B* **2020**, *1152*, 122276. https://doi.org/10.1016/j.jchromb.2020.122276.

(10) Förlin, L.; Sundelin, B.; Gorokhova, E.; Magnusson, M.; Bergkvist, J.; Parkkonen, J.; Larsson, Å.; Liewenborg, B.; Franzén, F. *Effektscreening – Biologisk Effektövervakning i Förorenade Områden Längs Sveriges Kust 2017–2018*; Nationell miljöövervakning på uppdrag av Naturvårdsverket.; 2019.

(11) Raymond, C.; Gorokhova, E.; Karlson, A. M. Polycylic Aromatic Hydrocarbons Have Adverse Effects on Benthic Communities in the Baltic Sea: Implications for Environmental Status Assessment. *Front. Environ. Sci.* **2021**, *9*. https://doi.org/10.3389/fenvs.2021.624658.

(12) Löf, M.; Sundelin, B.; Bandh, C.; Gorokhova, E. Embryo Aberrations in the Amphipod *Monoporeia Affinis* as Indicators of Toxic Pollutants in Sediments: A Field Evaluation. *Ecological Indicators* **2016**, *60*, 18–30. https://doi.org/10.1016/j.ecolind.2015.05.058.
